# Assemblages of Acari in shallow burials: mites as markers of the burial environment, of the stage of decay and of body-cadaver regions

**DOI:** 10.1007/s10493-021-00663-x

**Published:** 2021-10-07

**Authors:** Jas K. Rai, Brian J. Pickles, M. Alejandra Perotti

**Affiliations:** Ecology and Evolutionary Biology Section, School of Biological Sciences, University of Reading, Reading, Berkshire UK

**Keywords:** Biodiversity, Marker, Forensic, Shallow grave, Acarines, *Sus scrofa domesticus*

## Abstract

The burial of a cadaver results in reduced arthropod activity and disruptions in colonisation patterns. Here, the distribution and diversity of mite taxa was studied across decomposition stages of shallowly buried pig carcasses (*Sus scrofa domesticus*). In total 300 mites (88 species) were collected from three pig shallow graves compared to 129 mites (46 species) from control (bare) soil samples at the same depth. A successional pattern of Acari higher taxa and families was observed, and species richness and biodiversity fluctuated throughout decomposition, whereas active decay showed the greatest biodiversity. Mesostigmata mites were the most abundant in ‘cadaver soils’ with a significant difference in the abundance of Parasitidae mites, whereas Oribatida mites (true soil mites) were the most abundant in control soils. Certain mite species were significantly associated with decay stages: *Cornigamasus lunaris* with ‘bloated’, *Gamasodes spiniger* with ‘active’, *Eugamasus* sp. and *Lorryia reticulata* with ‘advanced’, and *Macrocheles matrius* and *Ramusella clavipectinata* in ‘dry’. *Scheloribates laevigatus* was a marker of bare soil at a shallow depth and *Vulgoramasus remberti* of buried decomposition, not specific to any decay stage. Analysis of mite assemblages associated with head, torso and posterior body showed that *Parasitus evertsi* and *M*. *matrius* are attracted to beneath the thighs, whereas *L*. *reticulata* to beneath the head. This study highlights the value of mites as indicator species of decomposition and its stages, confirming (1) a succession of Acari on buried remains and (2) species specificity to body regions.

**Supplementary Information:**

The online version contains supplementary material available at 10.1007/s10493-021-00663-x.

## Introduction

The burial of a cadaver results in interrupted insect activity and colonisation patterns, making it difficult to use insect evidence for time of death estimations. In such cases, evaluation of the composition and successive patterns of colonisation by the soil mite taxa can become more valuable. Mites can detect and colonise a cadaver by walking from the surrounding soil environment, inadvertently through anemochory, and also via phoresy with necrophagous Diptera and Coleoptera and mammalian scavengers (Perotti et al. 2009). The majority of studies on the acaro-fauna (mites) of cadavers are focused on surface decomposition whilst the mite fauna of buried remains is relatively unexplored. However, collectively these studies have demonstrated that as the soil environment undergoes continuous physical and chemical changes correlated to each phase of decay, the abundance and diversity of mites in the surrounding soil changes and faunal succession of mite species occurs.

Just a handful of studies have reported an abundance of Mesostigmata, Astigmata, and Oribatida mites from shallow and deep graves. Most of the early research on graves were focused on the fauna of coffin burials. In the late nineteenth century, Jean Pierre Mégnin recognised the importance of mites in the fauna of buried and exposed cadavers, and reported their presence during four waves of arthropod colonisation of buried bodies (Mégnin 1894). Murray Galt Motter in (1898) further developed the study of the mite fauna of graves and documented high abundances of 8 Mesostigmata and Astigmata species, along with insects, from 30 human bodies undergoing different decay stages, where *Uropoda depressa* (Mesostigmata, Uropodidae) was the dominant species (Motter 1898). More recent investigations of coffin burials have also reported the occurrence of mites during early decay stages (Bourel et al. 2004; Mariani et al. 2014) to late stages (Mariani et al. 2017; Merritt et al. 2007).

Assemblages of *Tyrophagus putrescentiae* (Astigmata) and Mesostigmata species (*Macrocheles merdarius*,* Paechylaelaps* sp., Uropodidae sp. and *Glyptholaspis americana*) were recovered from a clandestine human grave in soil during advanced decay and facilitated in post-mortem interval (PMI) estimations (Goff 1991). Likewise, the Macrochelidae species *Macrocheles glaber* and several Parasitidae species were colonising heavily decomposed human remains in a shallow grave, providing indications on the stage of decay and supporting the PMI estimated by flies (Kamaruzaman et al. 2018). Additionally, *Parasitus loricatus* (Mesostigmata, Parasitidae) and *Rhizoglyphus robini* (Astigmata, Acaridae) were recovered from buried human remains in advanced decay, and the Parasitidae species *Gamasodes spiniger* was recovered from buried human remains during active decay (Rai et al. 2020). An abundance of *Macrocheles* species was recovered from buried pig cadavers during active decay (Rysavy and Goff 2015) and Astigmata species (*Caloglyphus* sp.) were found feeding on desiccated tissue of buried pigs (Payne et al. 1968).

As cadaver decomposition occurs, soil chemistry significantly changes, which effects the abundance of soil organisms. Much of the regular mite fauna of soil is thought to disperse or die on introduction of a cadaver (Bornemissza 1957). In particular the pH of the soil beneath a cadaver fluctuates throughout the decay process and can become moderately to strongly alkaline (Carter et al. 2007). This has a direct impact on the abundance and diversity of the soil mites. For example, alkaline conditions are more favorable for Mesostigmata mites whilst acidic conditions are more favorable for Oribatida mites (Maraun and Scheu et al. 2000). Oribatida mites are soil-dwelling mites and are the dominant soil micro-arthropods in most soil types, particularly in forest soils where there may be up to 500,000 individuals/m^2^ (Schatz and Behan-Pelletier 2008). Oribatida mites are known as secondary decomposers in soil ecosystems and are mainly bacterial or fungal feeders that are associated with nutrient cycling of plant litter (Behan-Pelletier 1999; Krantz 1978). They are usually abundant in the soil during the fresh stage of cadaveric decay in forest habitats (Anderson and VanLaerhoven 1996) though their abundance in the soil is expected to decrease soon after decomposition progresses following the arrival of predatory Mesostigmata species (Perotti et al. 2009; Silahuddin et al. 2015). For example, shortly after decomposition of exposed guinea carcasses, a decrease in abundance of incidental soil dwelling Oribatida mites was observed along with a significant increase in other mites, such as Tyroglyphidae (Astigmata) (Bornemissza 1957). This change in soil mite abundance was mostly noticeable in the soil beneath the anal and mouth parts. In addition, the effect of decomposition on mite abundance was evident up to 14 cm in depth. During early stages of decomposition, it is likely that the seepage of decay fluids into the soil has a negative effect on Oribatida which may reappear during late (dry) decay when the soil begins to normalise (Goff 1989; Mariani et al. 2014).

Mesostigmata mites become the predominant mite group as decomposition begins, as the majority are predatory species extensively involved in decomposition processes of carrion in forest soils (Jung et al. 2010; Perotti and Braig 2009). Predatory soil-dwelling and phoretic Mesostigmata species exploit the carcass for food, for example, Parasitidae and Macrochelidae mites predate on Collembola, nematodes, insect larvae and other mites such as oribatids, already colonising the remains or occupying the surrounding soil (Koehler 1999). Several studies have recorded mites in association with the decomposition of vertebrate cadavers, including human’s, in terrestrial habitats as well as indoors, and the majority found that Mesostigmata mites are most abundant and diverse and are therefore considered to have forensic significance (Goff 1989, 1991; González-Medina et al. 2013; Kamaruzaman et al. 2018; Rysavy and Goff 2015; Saloña-Bordas and Perotti 2014, 2019).

Mesostigmata species are mostly free-living, and many disperse phoretically with insect and mammal hosts, consequently most of the phoretic mites associated with exposed carcasses are Mesostigmata (Barton et al. 2014; Perotti and Braig 2009; Perotti et al. 2009). Buried cadavers will also be colonized by phoretic mites; they will arrive with a few insect species specialized in burrowing into the soil to reach an interred cadaver. For example, species of Phoridae (Diptera) and Staphylinidae (Coleoptera) species will bring along an abundance of phoretic mites to a buried carcass, such as *Macrocheles* sp., *Parasitus* sp. and *Gamasodes* sp. (Fain and Greenwood 1991; Hyatt 1980; Perotti and Braig 2009). Up to now Macrochelidae mites represent the greatest number of phoretic mites known to be associated with exposed cadavers, they are phoretic and may be transported with the primary colonising Diptera in the first wave of arthropod colonisation of surface cadavers (Barton et al. 2014; Kamaruzaman et al. 2018). They are followed by *Parasitus* and/or *Poecilochirus* (Parasitidae) as well as other macrochelids carried by beetles in later stages (Kamaruzaman et al. 2018).

Astigmatid mites, such as Acaridae and Histiostomatidae species also inhabit soils. Many Astigmata species are associated with carrion and may be found during dry stages of cadaver decay (e.g., skeletonisation or mummification) in exposed and buried cadavers (Early and Goff 1986; Merritt et al. 2007; Motter 1898; OConnor 2009; Payne 1965; Payne et al. 1968; Russell et al. 2004). Astigmatids may be incidental soil-dwelling species or necrophages that arrive phoretically with insects and therefore may even be present in the first wave (OConnor 2009; Saloña-Bordas and Perotti 2020). Prostigmata mites are microbial and plant feeders in soil habitats, whereas some species are predaceous; however, the incidence of Prostigmata mites occurring in graves is entirely unknown though they have been recovered from exposed cadavers (Braack 1986).

The burial of a cadaver results in reduced insect activity and disruptions in colonisation patterns, hence the need to develop new methods for analysis of the ubiquitous soil fauna in soils, that might associate with buried bodies. Both the distribution of mite taxa across decomposition stages—and the affected soil pH—and the identity of forensically important mite species of buried cadavers are entirely unknown. Focusing on the decomposition stages of shallowly buried pig carcasses the aims of this study were: (1) to determine the impact of decay and changes in soil pH on the abundance, diversity and successional patterns of mite taxa; (2) to identify mite species that may be indicators of decomposition stages and any incidental mite species that may be indicators of bare soil at the same depth within the soil; and (3) to identify whether body regions of decomposing cadavers are associated with specific assemblages of mite species.

## Materials and methods

### Experimental design

The study was carried out in a relatively undisturbed temperate woodland region at the University of Reading (Reading, Berkshire, UK; 51*°*26′10.6″N, 0*°*56′35.0″W). This area is an experimental ground located within the Whiteknights campus, a forested site situated beside a road. It is closed off to public access by a border of wire fencing and high foliage. It is approximately a rectangular area of 80 m long × 50 m wide, with vegetation consisting of a variety of deciduous trees and shrubbery, of relatively high canopies simulating a common ‘dumping’ site of a homicide victim by a perpetrator.

Three freshly killed pig carcasses (*Sus scrofa domesticus*) were used as human proxies. Previous research recommends a pig carcass of 23 kg as a minimum appropriate weight (Catts and Goff 1992). A larger size does not significantly affect the pattern of decay and arthropod succession (Hewadikaram and Goff 1991). The pig carcasses (n = 3) were studied over a period of 3 years from 3rd October 2015 until 30th September 2018, with 1 pig cadaver studied each year consecutively, for approximately 1 year each (P1–P3; Table 1). The pig cadavers used were purchased at a slaughterhouse. Each carcass was immediately placed into secured body bags to prevent contamination by insects and transported to the study site. Their use on University grounds (Whiteknights Campus), and the transport from the abattoir to the University of Reading followed regulations established by the Animal Health and Veterinary Laboratories Agency (AHVLA), after registration: ‘ABP Registration Reference: U1116918 Notification of registration for the generation, transportation, handling, processing, storage, placing on the market, distribution, use or disposal of animal by ~ products (ABP’s) or derived products under the requirements of Article 23 of Regulation (EC) No. 1069/2009’.

**Table 1:** Details of each of the three pig carcass subjects

Subject	Weight (kg)	Control soils	Grave dimensions (cm)	Depth of topsoil (cm)	Date of placement	Date of study termination	Period of study (days)
P1	30	C1	82 × 51 × 10	3	6 Oct 2015	30 Sep 2016	364
P2	30	C2	126 × 76 × 22	4	12 Oct 2016	21 Mar 2018	526
P3	40	C3	96 × 32 × 22	3	3 Oct 2017	30 Sep 2018	363

A grave was prepared for each study with depths of 10–22 cm and with varying grave dimensions (depending on the size of the carcass) (Table 1). Each carcass was placed in its grave during the first 2 weeks of October of each year and studied until the carcass was reduced to only bones. Each grave site was positioned at least 10 m away from the previous grave site. Corresponding control soils plot areas (C1–C3) were marked for collection of control soils (bare soil) for each cadaver studied which were at least 20 m away from the graves with at least 10 m between two control soil plots.

**Fig. 1:**
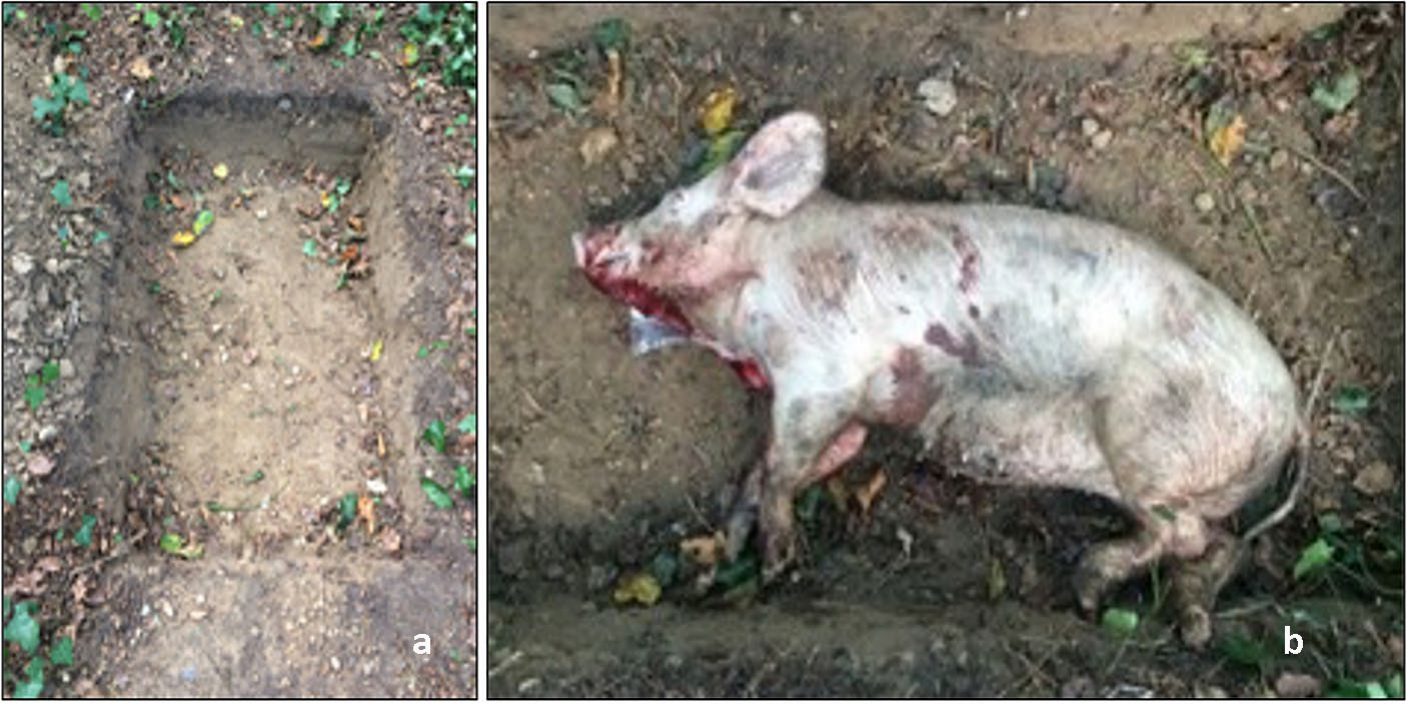
Example of excavated grave used for pig cadavers. **a** Grave pit for pig cadaver 2 (P2), of approximately 126 × 76 × 22 cm. **b** P2 placed laterally within the grave

Each carcass was placed laterally and directly on the soil within the grave (Fig. 1). The pigs were covered with the soil and plant debris that was removed to form the grave and was left loose. The depth of the topsoil covering each pig carcass was approximately 3–4 cm. Each grave was enclosed with a mesh metal wire cage (110 × 140 × 60 cm, mesh thickness 0.5 cm) to deter vertebrate scavengers from feeding on the carcass whilst allowing invertebrate scavengers free access.

### Decomposition stages

The decomposition process of the pig cadavers was assessed visually 3–5 times a week. The post mortem changes were recorded and the decomposition process was divided into the five major stages: fresh, bloated, active, advanced and dry/remains. The fresh stage was characterised by initial post mortem changes such as *livor mortis* (purple discoloration of the skin) and *algor mortis* (decline in body temperature) which was evident within a few hours after death and prior to the burial. Internal build up volatile organic gases resulted in significant inflation of the cadavers (bloated stage) which caused the layer of loose soil covering the cadavers to be pushed away on some parts, exposing regions of the body surface to the atmosphere. This allowed egg laying by Diptera to take place directly on the exposed areas. The emerging dipteran larvae were observed feeding on the soft tissue of the exposed areas and along with the emittance of strong decay odours, this marked active decay. Advanced decay is characterised by the appearance of bones and the presence of some decay fluids from the breakdown of soft tissue during active decay. The dry/remains stage was characterised by the total body skeletisation and the presence of hardened skin, cartilage and hair.

### Soil sampling and extraction of mites

Soil samples of a 300-mL volume from around and beneath the head, abdomen and posterior region (300 mL from each region) were sampled from each pig carcass, giving a total soil sample volume of 900 mL on each sampling day. This soil is denoted as ‘cadaver soil’. To sample from beneath the carcass, a larger shovel was used to lift the carcass at an angle whilst a hand shovel was used to retrieve soil from underneath. Sampling days were decided according to post-mortem changes of the carcass associated with each decomposition stage. Three soil samples per stage of decay (total of 15 soil samples) were collected and analysed for each cadaver (Table 2). The first soil sample was collected when the first physiognomies of that stage were evident, followed by a second soil sample when further changes were observed and then a final soil sample was collected when no further changes of that stage were observed. Control soils (bare soil) of the same volume were obtained from control soil plots approximately 10 m away (‘control soil’). The mites collected were grouped into each of the five stages of decay in order to assess the faunal succession associated with decomposition. The control soils were taken at the same depth as the corresponding grave studied and on the same sampling days and a total 900 mL of control soil was collected on each sampling day (3 × 300 mL).

**Table 2:** Days sampled post-mortem during each stage of decomposition of the three buried pig cadavers (P1–P3)

Stage	Sample nr	Sampling day P1 starting 6 Oct 2015	Sampling day P2 starting 12 Oct 2016	Sampling day P3 starting 3 Oct 2017
Fresh	1	1	1	1
	2	3	3	3
	3	4	7	5
Bloated	4	8	9	6
	5	17	15	15
	6	25	38	22
Active	7	38	44	38
	8	87	96	72
	9	101	131	149
Advanced	10	136	156	169
	11	169	233	222
	12	294	336	274
Dry/remains	13	336	365	302
	14	350	448	333
	15	364	526	363

Sampled soils were placed directly into zip lock plastic bags and transported to the laboratory. Soil samples were immediately placed into manually made Berlese-Tullgren funnels in an indoor laboratory for mite extraction, for a total of 7 days. In each Berlese-Tullgren funnel, 300 mL of soil was used for extraction from the soil from graves and the control soils—i.e., 300 mL from the head, abdomen and posterior region—was placed under three separate funnels and the 3 × 300 mL of control soils was placed into a further three separate funnels. The mite data were pooled together for each body region and for each stage in order to represent the mite abundance associated with each stage of decomposition.

A soil volume of 300 mL was selected for the mite extraction process with Berlese-Tullgren funnels based on a separate preliminary study of mite biodiversity in soil in the same area (Online Resource 1: Figs S1 and S2; Tables S1 and S2). In this preliminary study, the total number of mite species extracted via Berlese-Tullgren funnels from soil samples of volumes 100, 200, 300, 400, and 500 mL from beneath four pig cadavers placed on the surface and from four plots of bare (surface) soil, was assessed. A line curve was plotted showing the species richness of each soil volume and a plateau or decrease in biodiversity with increasing volume was observed after 300 mL, therefore, 300 mL was determined to be a suitable volume of soil for optimum mite extraction via Berlese-Tullgren funnels in this area of Berkshire, Reading. The duration of arthropod extraction by Berlese-Tullgren funnels can affect the diversity collected and longer time periods allow for slower moving micro-arthropods to fall into the collection vials. Most mite extraction generally occurs in the first 2 days; however, studies have recovered mites up to 7 days (Barberena-Arias et al. 2012; Søvik and Leinaas 2002). Incandescent light bulbs of 40 W were used to encourage mites, which respond negatively to heat and light (Tullgren 1918), to fall into collection vials below containing 70% (v/v) ethanol for preservation.

### Measurement of soil pH

To address mite taxa throughout the decomposition process and the consequent changes in soil pH, from each soil sample collected from both cadavers and controls, 10 g of soil was collected. It was weighed in a sterile weighing boat and mixed with 25 mL of deionised water in sterile containers at a ratio of 1:2.5 (soil: solution). The soil solution was left to settle at room temperature for 30 min and the pH was recorded using a Pentype Digital pH Meter (Tekcoplus, Hong Kong). This ratio of 1:2.5 was used as it is the most commonly suggested ratio for soil pH measurements (Aciego Pietri and Brookes 2008).

### Counting of mites

Mites were extracted from soil samples, separated and cleared using methods previously described (Krantz 1978). The content of the collection jars [containing 70% (v/v) ethanol and all arthropods from the Berlese-Tullgren funnels] was transferred into Petri dishes and mites were separated from all other arthropods under a stereo microscope. Other arthropods were checked for attached phoretic mites and any attached mites were manually removed. Individual mites were tallied and recorded manually, and mite abundances were separated into the five stages of decomposition of each pig cadaver and the corresponding control soils. After mites were counted, all specimens were transferred into Eppendorf tubes containing 50% lactic acid (v/v) solution and were left until soft tissues were macerated (cleared), allowing external and hard tissue structures to be clearly visible for the purpose of future mounting and taxonomic identification.

### Preparation and mounting of mite specimens

All cleared mites were mounted on glass slides using Hoyer’s medium based on methods previously described (Krantz 1978). The mites were covered with a glass cover slip and sealed with red enamel paint; Glyptal®, and was left to dry at room temperature for 24 h. Mite counts were made for each sample and data for head, body and posterior regions were pooled together for each sampling block.

### Taxonomic identification of mites

Mite specimens were identified using a phase contrast microscope with magnifications of 10–100 × (Nikon Optiphot). All mites were initially classified into four major mite groups: Mesostigmata (order within the Parasitiformes), Oribatida (suborder within the Sarcoptiformes), Astigmata (cohort within the Oribatida), and Prostigmata (suborder within the Trombidiformes) (Baker 1999; Krantz and Walter 2009).

The Mesostigmata were identified based on the presence of three major features: peritremes on side of the body ending in stigmatic openings, presence of a tritosternum and free and unfused leg coxae. The mites belonging to the Astigmata were identified based on the absence of stigmatic openings and leg coxae fused to the body forming apodemes. The Prostigmata were identified based on the presence of stigmatic openings associated with the chelicerae or on the upper region of the body (propodosoma), leg coxae fused to the body forming apodemes and in many cases a distinct wider upper part of the body compared to the lower part. The Oribatida were identified based on the level of sclerotization as most Oribatida mites are large and highly sclerotized with thick cuticles. Mites from each group were then identified and classified into families based on taxonomical keys and diagrammatic interpretations from the descriptions detailed by Krantz and Walter (2009).

Individuals were identified to the species level using numerous taxonomical keys on Mesostigmata (Evans 1956; Evans and Till 1979; Hennessey 1989; Hyatt 1980; Hyatt and Emberson 1988; Juvara-Bals and Witaliński 2006; Kazemi et al. 2013; Krantz and Walter 2009; Mašán and Halliday 2014; Özbek et al. 2015; Teodorowicz et al. 2012; Witaliñski 2005), Oribatida (Baker 1999; Krantz and Walter 2009; Michael 1884, 1888). Astigmata (Hughes 1976; Krantz and Walter 2009) and Prostigmata (Hughes 1976; Krantz and Walter 2009). In the infrequent circumstance where a mite was found broken and thus unidentifiable, the specimen was identified to the order level (if possible) and labelled as an ‘unknown species’. Within these ‘unknown species’, the mites were observed for any basic similar or identical morphologies that were visible. If similar or identical morphologies were identified between individual mites, they were categorised into a morpho-species and coded with a number under that group, for example, ‘Mesostigmata unknown species 1, 2, 3’ etc. For any single mite that did not bear any similarities with any other individual, it was categorised into its own morpho-species.

### Data analysis

Various exploratory techniques (bar charts and line graphs) were produced in Excel 2019 and Minitab® v.19.2020.1.0 to visualise the data of mite taxa abundances. For all statistical analyses we used a significance threshold of α = 0.05.

The significance of any changes in soil pH during decomposition was tested using separate Generalized Linear Models for each replicate with treatment (cadaver, control), and decay stage (five levels) as interactive predictor variables in R v.4.0.2. The extracted mites were grouped into the five decay stages in order to assess the abundances and diversity of different mite groups associated with stages of decomposition of cadavers and control soils. Within each decay stage, mites were identified and grouped into the four major mite taxa (Mesostigmata, Oribatida, Astigmata and Prostigmata) and then classified into mite families, and the significance of any difference in abundances of each mite higher taxon and mite family between grave soil, control soils and stages of decomposition was tested with the non-parametric Kruskal–Wallis test (adjusted ‘for ties’), conducted in Minitab v.19.2020.1.0. The species diversity of mites associated with each decomposition stage was explored in terms of species richness (S), Shannon-diversity indices (H), and species evenness (E) conducted in PAST v.4.03 (Hammer et al. 2001). Multivariate Indicator species analysis using Monte Carlo significance testing with 999 randomisations (Dufrêne and Legendre 1997), was used to identify mite species as forensic markers of: (1) cadaver soil compared to control soil, (2) decomposition stages in cadavers compared to control soils, and (3) body regions of pig cadavers and the corresponding control soils. Null hypotheses were tested in relation to identifying mite species as indicators of soil associated with decomposition and control (no decomposition) soil, individual cadavers, individual control soils, decay stages and body regions of cadavers. This analysis was conducted with PC-Ord v.5.0 (Wild Blueberry Media, Corvallis, OR, USA).

## Results

### Soil pH

**Fig. 2:**
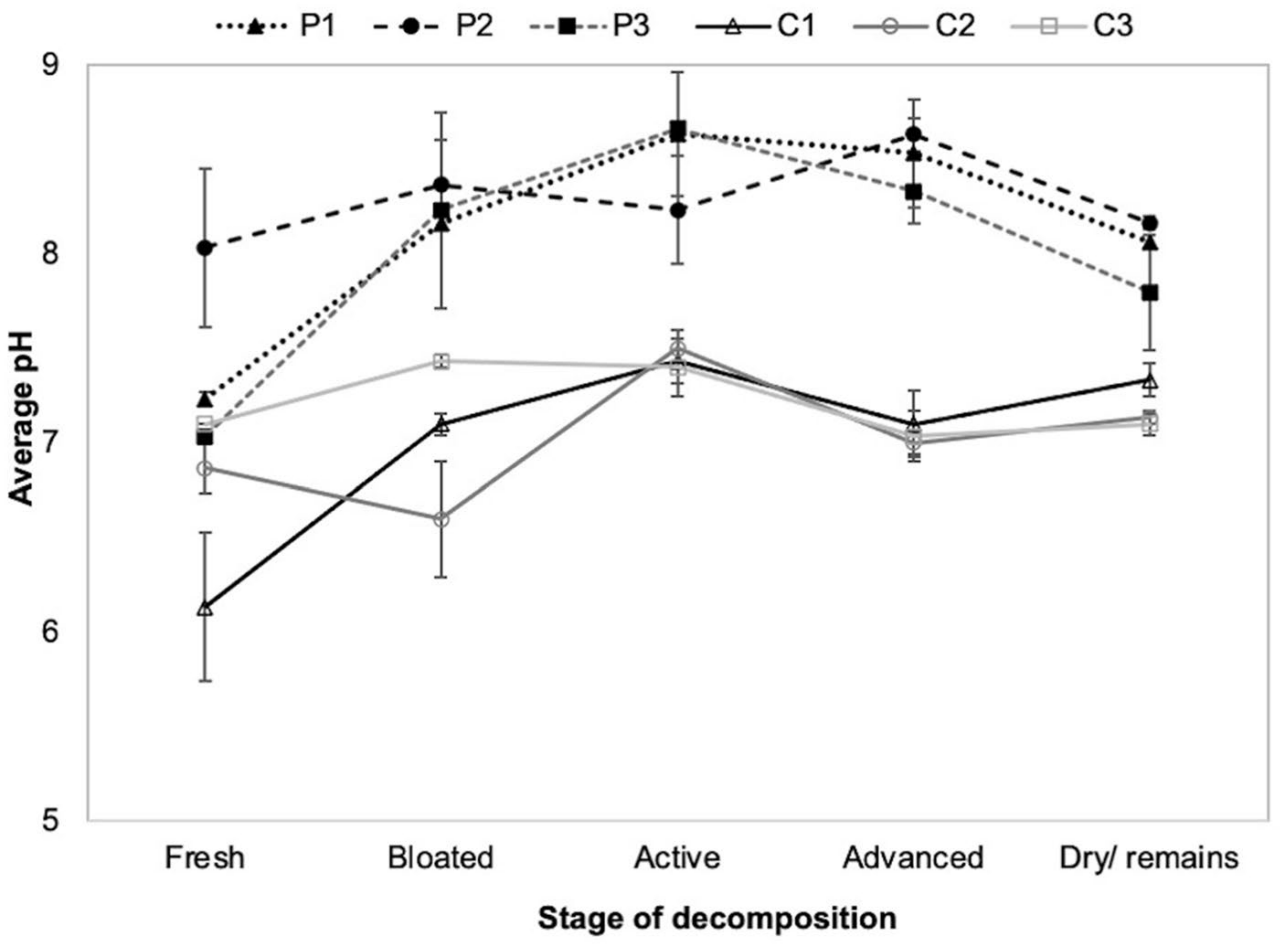
Mean (± SE; n = 3) pH of soil from each pig cadaver (P1–P3) during successive stages of decomposition and the corresponding control soils (C1–C3)

In cadaver soils, pH rose from an initial 7.1–7.3 at the time of burial to 8.1–8.8 after 8 days. The pH of the soil from all three pig cadavers fluctuated throughout decomposition, changing from neutral during early decomposition to moderately and strongly alkaline during mid to late decomposition, whereas the pH of the three control soils remained relatively and consistently neutral throughout the experiment (Fig. 2; Online Resource 2: Table S3). In replicates 1 and 2, mean soil pH was higher in cadaver soils than in control soils throughout the experiment (GLM_Rep1_ *t* = 3.62, P = 0.002; GLM_Rep2_ *t* = 2.12, P = 0.047), with replicate 1 showing significantly higher pH in the bloated, active, and advanced stages than the fresh and dry stages (Fig. 2; Online Resource 2: Table S4). In replicate 3, mean soil pH was significantly higher in the cadaver soils only during the bloated, active, and advanced stages (Fig. 2; Online Resource 2: Table S4).

### Successional patterns of mite groups during decomposition stages

**Fig. 3:**
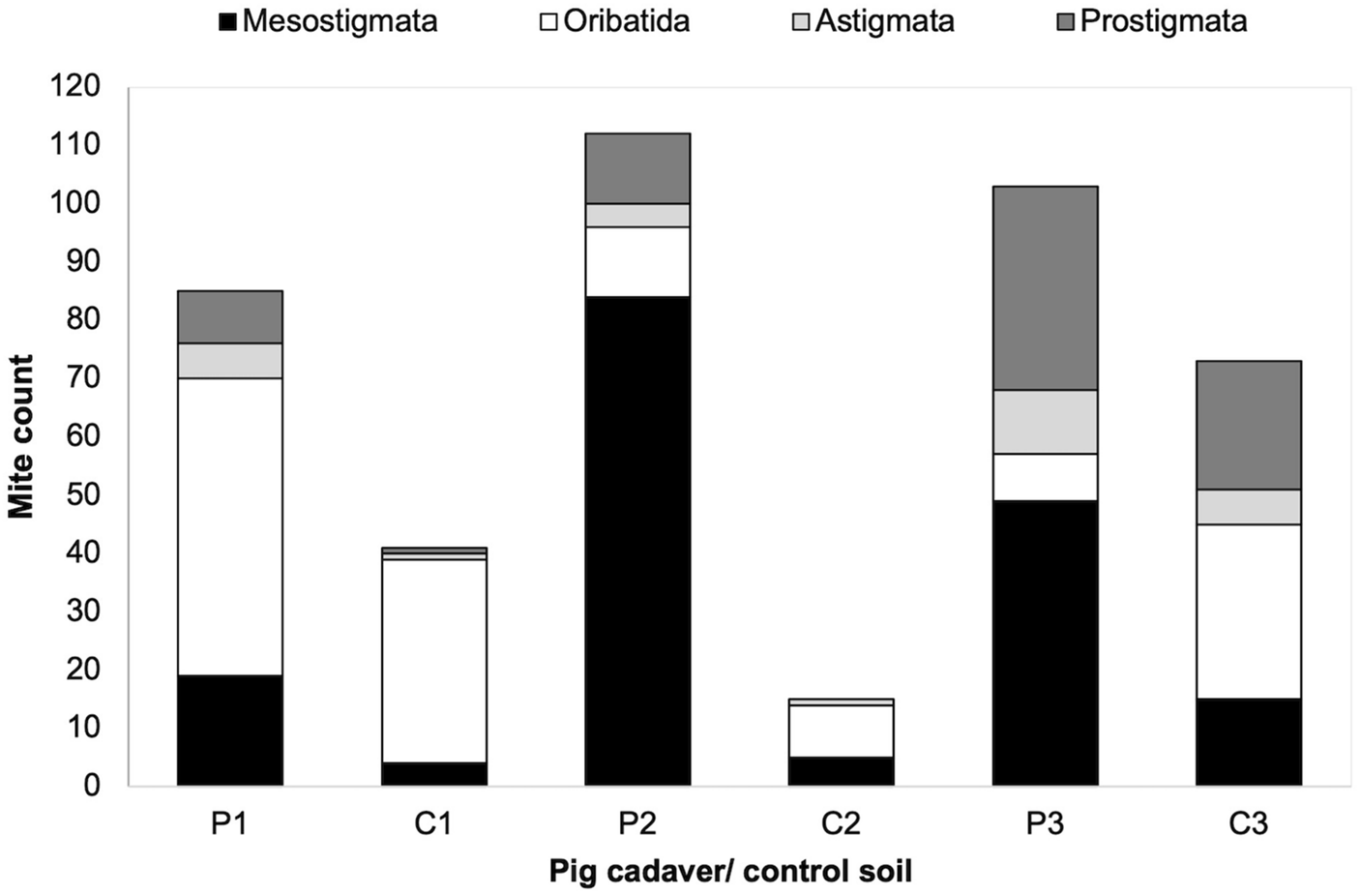
Abundance of mites belonging to four major mite groups from grave soil of pig cadavers (P1–P3) and corresponding control soils (C1–C3)

Mites extracted from all soil samples belonged to the four main mite groups: Mesostigmata, Oribatida, Astigmata and Prostigmata. Overall, Mesostigmata mites were the most abundant from all three cadavers giving a total of 152 mites, followed by Oribatida with 71 mites, Prostigmata with 56 mites, and Astigmata with 21 mites. In the control soils, Oribatida was the most abundant mite group with a total of 74 mites, followed by Mesostigmata with 24 mites, Prostigmata with 23 mites, and Astigmata with eight mites (Fig. 3).

The abundance of Mesostigmata, Oribatida, Astigmata and Prostigmata mites fluctuated throughout decomposition of all cadavers and control soils. There was an overall increase in abundance of all mite groups as the cadavers progressed from fresh to mid-decomposition (Fig. 3). Mesostigmata mites saw the largest increase in abundance compared to other mite groups for all three cadavers. There was a slight decrease during active and advanced decomposition followed by an increase during the dry stage; but in general, no significant difference was observed in abundance of Mesostigmata mites between stages of decomposition, when comparing all carcasses (Kruskall-Wallis, H = 5.88, d.f. = 4, P = 0.12) (Online Resource 3: Table S5).

**Fig. 4:**
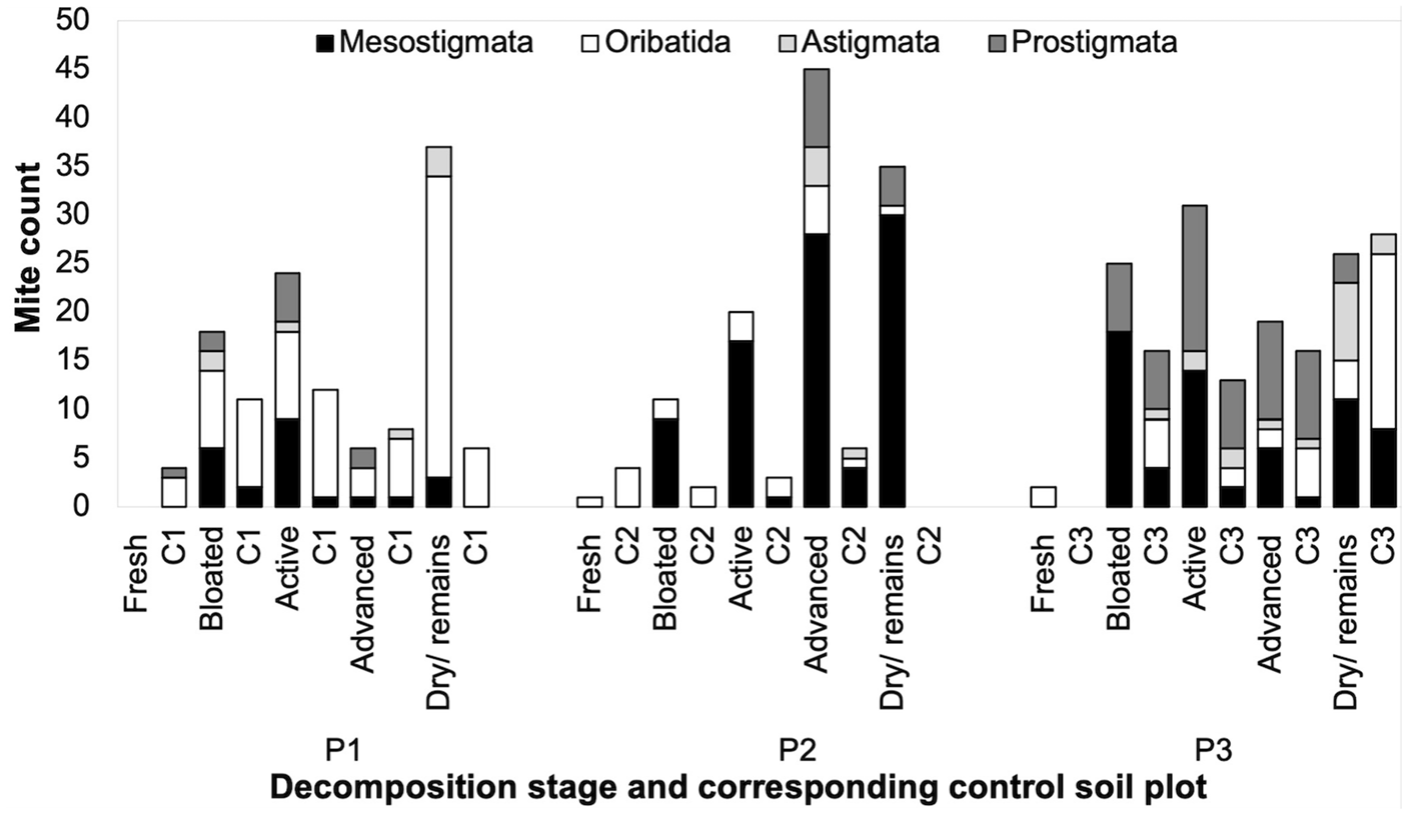
The total abundance of Mesostigmata, Oribatida, Astigmata and Prostigmata mites during fresh, bloated, active, advanced and dry/remains decomposition of cadavers (P1–P3) and the corresponding control soil plots (C1–C3) collected during successive stages of decomposition

No significant difference was observed in the abundance of Oribatida mites between stages of decay (H = 2.53, d.f. = 4, P = 0.55) (Online Resource 3: Table S5). Oribatida were found throughout decomposition in relatively low numbers from P2 and P3 but were comparatively more abundant from P1. They especially peaked during the dry stage of P1. Oribatida mites were less abundant in P3, and were entirely missing during bloated and active stages from this cadaver. Astigmata mites were overall the least abundant group of mites for all three cadavers. The highest abundance was during the dry stage in P3, and there was no significant difference between stages of decay (H = 4.19, d.f. = 4, P = 0.38) (Online Resource 3: Table S5). Prostigmata mites appeared to be mainly occurring during mid decomposition of all cadavers (bloated-advanced); despite this, no significant difference was observed between stages of decomposition (H = 5.81, d.f. = 4, P = 0.21) (Online Resource 3: Table S5). In relation to control soils, Oribatida were the most abundant group of mites during the dry stage for control C1 and during fresh and bloated stage for C2 (only contained Oribatida mites). Interestingly, for C3, during bloated, active and advanced Prostigmata were the dominating mites (Fig. 4).

### Successional patterns of mite families associated with decomposition stages

In total 26 mite families were identified in cadaver soils and 23 in control soils. There was a clear pattern of succession of mite families throughout decomposition of pig cadavers (Table 3), compared to corresponding control soils (Online Resource 4: Table S6). Overall, in all pig cadaver soils, eight Mesostigmata families were identified. The predominant Mesostigmata family in terms of number of individual mites was the Parasitidae, with a total of 68 mites collected throughout decomposition of all cadavers. The occurrence of Parasitidae mites appeared to be concentrated during bloated, active, and advanced decomposition with the greatest abundance during advanced decomposition (Table 3). Macrochelidae was the second most abundant family in terms of individuals with a total of 45 mites and appeared to be most strongly associated with the dry/remains stage of decomposition (Table 3). In total nine Oribatida mite families were collected from cadaver soils. Overall, Quadroppiidae and Oppiidae were the two most abundant Oribatida families. Quadroppiidae was associated with the highest number of total individuals (20 mites) collected during every stage except the fresh stage, but mostly during active and dry/remains. Oppiidae was the second most abundant with a total of 17 mites, but they were only recovered during dry decomposition (Table 3). In total, five Astigmata families and relatively low mite numbers (between 0 and 5 individuals) of each family occurred throughout decomposition. There did not appear to be any strong associations of Astigmata mites with any stages of decomposition in these shallow graves (Table 3). In total five Prostigmata families were recovered, whereby Tydeidae appeared to be associated with mid-decomposition stages, peaking in abundance during advanced decomposition. For the Mesostigmata families Diathrophallidae and Uropodidae only two individual mites were identified from each.

**Table 3:**
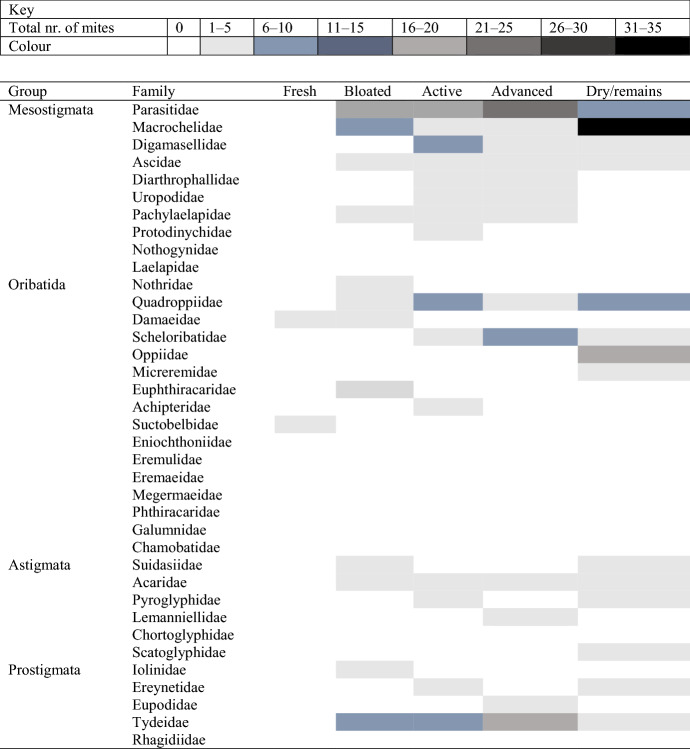
Successional patterns of mite families collected throughout the five stages of decomposition of the pig cadavers (n = 3)

In the control soils the abundance of mite families appeared to fluctuate throughout the study period; however, no succession was evident, as expected (Online Resource 4: Table S6). The number of Mesostigmata families was much lower (only six collected), whereas Oribatida were numerous (13 families collected). The most represented was the Quadroppiidae, with a total of 20 mites occurring throughout the full collection time period and secondly Scheloribatidae (Oribatida) (19 mites). In total four Astigmata families were collected from control soils (carrying just 0–5 individuals) and one Prostigmata family, Tydeidae. Several mite families that were found in cadaver soils were not recovered from the control soils, these include: (Mesostigmata) Digamasellidae, Diathrophallidae, Uropodidae and Protodinychidae; (Oribatida) Nothridae, Achipteridae and Suctobelbidae; (Astigmata) Lemanniellidae and Scatoglyphidae; and (Prostigmata) Iollinidae, Ereynetidae, Eupodidae and Rhagiidae.

The five most abundant mite families in cadaver soils were Parasitidae, Macrochelidae, Quadroppiidae, Tydeidae and Oppiidae (> 17 individuals) where all other families had fewer than 11 individuals. Kruskal–Wallis tests (adjusted for ties) indicated that the abundance of Parasitidae mites across decomposition stages was significantly different (H = 9.60, d.f. = 4, P = 0.04) but the abundance of Macrochelidae mites across decomposition stages was not (H = 7.96, d.f. = 4, P = 0.09). Likewise, there was no difference in abundance of Quadroppiidae (H = 3.15, d.f. = 4, P = 0.53) as well as for Tydeidae mites between decomposition stages (H = 6.27, d.f. = 4, P = 0.18) (Online Resource 5: Table S7). The abundance of Oppiidae between stages was not tested as they were solely found during the dry stage.

### Species richness and diversity by stages of decomposition

**Fig. 5:**
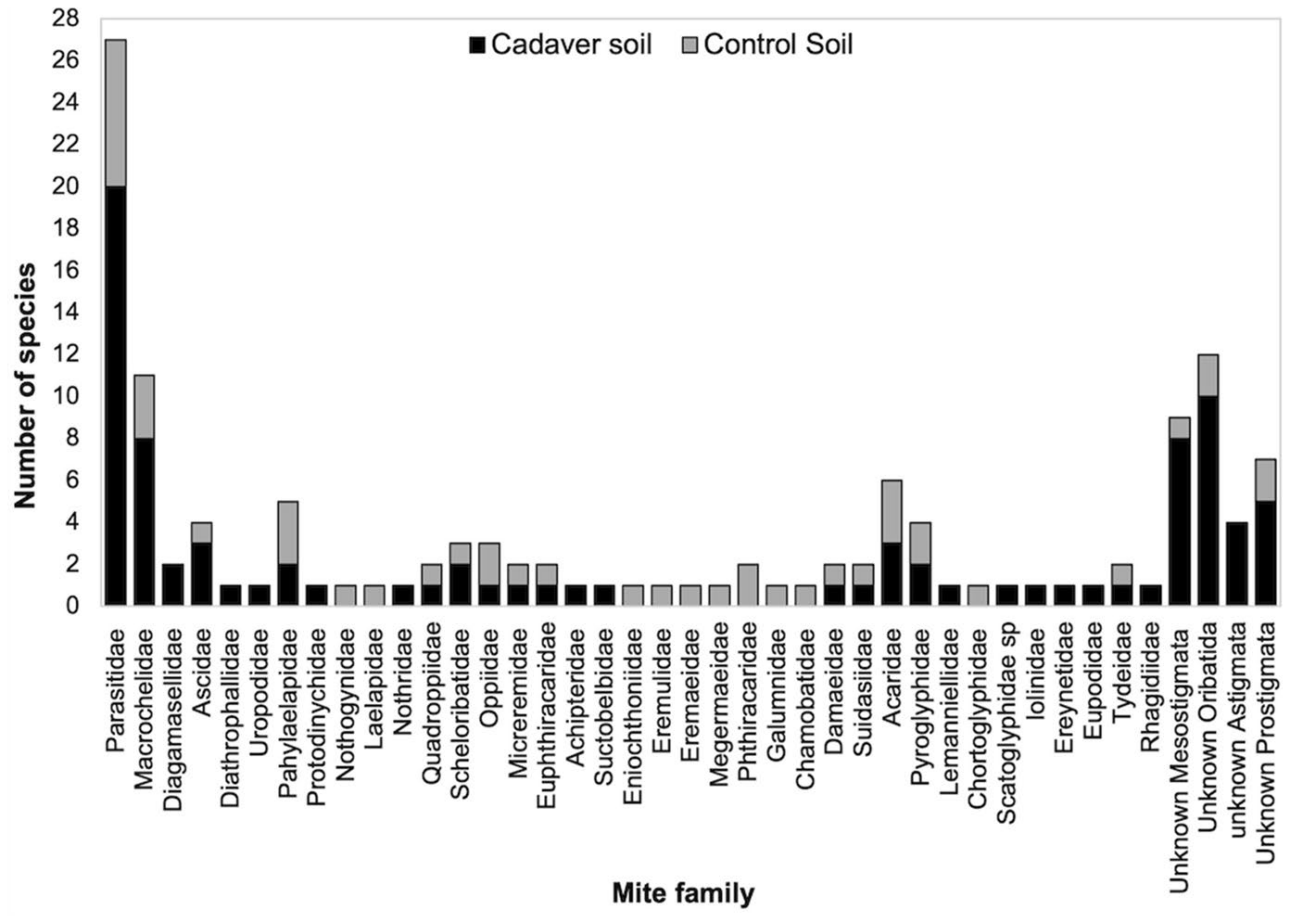
Total number of species belonging to mite families collected throughout decomposition of pig cadavers (n = 3) and corresponding control soils

From all three pig cadavers, in total 88 mite species were identified. Additionally, 46 mite species were identified throughout the study from all control soil plots. In the cadaver soils, the most species-rich family was Parasitidae with a total of 20 species compared to seven found in control soils. This was followed by Macrochelidae, with eight species collected from cadaver soils and three from control soils. Other mite families were associated with relatively low numbers of species in cadaver soils (1–3 species). Most species from control soils belonged to Oribatida families (Fig. 5).

The overall species richness, evenness and diversity fluctuated over the course of cadaver decomposition (Online Resource 6: Table S8). Overall, active decomposition was associated with the greatest species richness (S = 38) and diversity as represented by Shannon diversity index (H = 3.27) with an evenness (E) of 0.69. The bloated stage of decomposition was associated with the second greatest species diversity (H = 2.96) with a total of 27 species where the species evenness was greater (E = 0.711). Advanced decomposition had a slightly lower diversity (H = 2.77) with a total of 27 species; however, a lower evenness than the bloated stage of 0.59. The dry/remains stage had a greater number of species than bloated and advanced (S = 30); however, it was represented by the lowest evenness (0.43), demonstrating that the 30 species collected during the dry stage showed the highest differences in their relative abundances. The diversity was lower (S = 2.57) compared to the bloated and advanced decomposition. The fresh stage was associated with the lowest number of species (S = 2) and diversity (H = 0.637) with the highest evenness of species (E = 0.94) showing that the two species collected showed the lowest differences in their relative abundances. In comparison, the greatest diversity in the control plots was in the control soils collected during active decay (H = 2.62) with an evenness of 0.86, whereas the lowest diversity associated with the control soils was when the cadavers were undergoing fresh stage (H = 1.49) with an evenness of 0.89 (Online Resource 6: Table S9).

### Mite species as indicators or markers of decomposition stage, control soil, and body regions

The observed distribution of species incidence and abundance were examined and compared to a 999 randomised community draw from the same species pool. In total 13 mite species were identified as significant markers, eight were Mesostigmata species, four Oribatida, and one Prostigmata; no Astigmata species were identified as marker of the shallow burial environment.

**Fig. 6:**
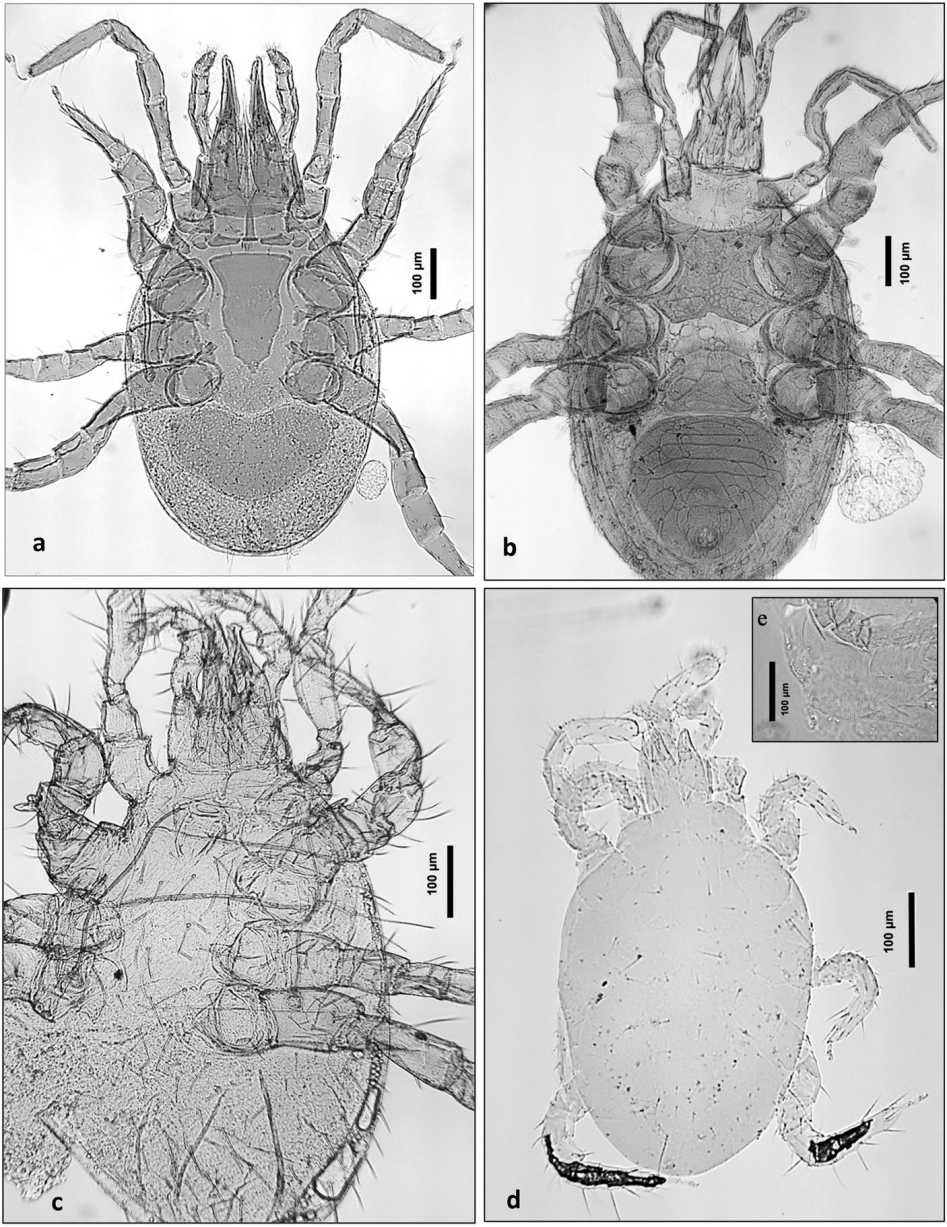
Major mite species markers of graves. **a** *Cornigamasus lunaris*. **b** *Macrocheles matrius*. **c** *Vulgarogamasus remberti*. **d** *Lorryia reticulata*. **e** Inset showing the characteristic fine reticulations on the body of *L*. *reticulata* (ventral)

Four mite species were significant indicators for cadaver soils, i.e., significantly associated with decomposition rather than with bare soil (Table 4, Contrast 1): *Cornigamasus lunaris* (Fig. 6a), *Macrocheles matrius* (Fig. 6b), *Vulgoramasus remberti* (Fig. 6c) and *Lorryia reticulata* (Fig. 6d, e). One species was found to be an indicator specifically for control soil: *Schleroribates laevigatus* (Table 4, Contrast 1). *Lorryia reticulata* was more abundant in pig cadaver P3 (Table 4, Contrast 2), whereas *Quadroppia michaeli* was significantly more abundant in pig cadaver P1 (Table 4, Contrast 2) and control plot C1 (Table 4, Contrast 3).

**Table 4:** Indicator analysis showing those mite species with significantly higher abundance and/or incidence between contrasting groups of observations (1–7)

Contrasts	Mite species	Group	Observed indicator value	Monte Carlo mean	Monte Carlo SD	P*
1. Cadaver vs. control	*Cornigamasus lunaris*	Cadaver	4.4	2.1	0.78	0.02
	*Vulgarogamasus remberti*	Cadaver	4.4	2.1	7.9	0.04
	*Macrocheles matrius*	Cadaver	7.3	3.4	1.13	0.01
	*Scheloribates laevigatus*	Control	9.2	6.3	1.35	0.03
	*Lorryia reticulata*	Cadaver	9.4	5.7	1.31	0.01
2. Between cadavers (P1-P3)	*Quadroppia michaeli*	P1	31.6	11.7	4.94	< 0.01
	*L*. *reticulata*	P3	39.6	17.5	5.82	< 0.01
3. Between controls (C1-C3)	*Q*. *michaeli*	C1	48.0	16.7	5.91	< 0.01
4. Between decay stages	*C*. *lunaris*	Bloated	40.0	13.8	7.45	0.02
	*Gamasodes spiniger*	Active	29.6	11.2	6.26	0.03
	*Eugamasus* sp.	Advanced	33.3	10.0	6.70	0.03
	*L*. *reticulata*	Advanced	28.6	16.2	6.07	0.04
	*M*. *matrius*	Dry/remains	38.3	13.0	6.93	0.01
	*Ramusella clavipectinata*	Dry/remains	33.3	11.6	6.68	0.04
5. Between cadaver × decay	*R*. *clavipectinata*	P1, dry/remains	62.7	24.9	10.16	< 0.05
	Mesostigmata sp. C	P2, advanced	66.7	18.7	9.83	0.04
	*Eugamasus* sp.	P2, advanced	100.0	17.4	10.90	< 0.01
6. Between control × decay	*Pachylaelaps longisetis*	C2, advanced	66.7	18.7	9.94	0.04
	*L*. *reticulata*	C3, bloated	44.4	16.7	10.67	0.04
7. Between regions	*Parasitus evertsi*	Posterior	6.7	1.8	1.34	0.03
	*M*. *matrius*	Posterior	6.7	3.5	1.72	< 0.05
	*L*. *reticulata*	Head	11.1	4.0	1.42	< 0.01
	*Q*. *michaeli*	Control, head	7.3	3.8	1.57	0.03
	*S*. *laevigatus*	Control, head	11.0	4.2	1.46	< 0.01
	*Hoplophora anomala*	Control, head	7.4	2.2	1.21	0.01

*Proportion of 999 randomized trials with indicator value equal to or exceeding the observed indicator value. *P* = (1 + number of randomized runs ≥ observed)/(1 + number of randomized runs)

Comparisons of individual mite species between decay stages in pig cadavers showed that *C*. *lunaris* was an indicator for the bloated stage, *Gamasodes spiniger* was a marker for active decay, both *Eugamasus* sp. and *L*. *reticulata* were indicators of advanced decomposition, and both *M*. *matrius* and *Ramusella clavipectinata* were indicators for the dry stage of decomposition (Table 4, Contrast 4). Analysis of mite species between the sampled ‘stages’ of corresponding controls revealed no significant differences (all species P > 0.05).

Examination of the interactions between individual pigs and decay stages revealed that *R*. *clavipectinata* was a significant indicator for the dry stage of decomposition in pig cadaver P1, whereas *Eugamasus* sp. and Mesostigmata sp. C (unidentified species) were indicators for advanced decomposition of pig cadaver P2 (Table 4, Contrast 5). In the corresponding control samples, *Pachylaelaps longisetis* was an indicator for control plot C2 during the period when pig cadaver P2 was undergoing advanced decomposition, and *L*. *reticulata* was an indicator for control plot C3 during the period when pig cadaver P3 was in the bloated stage of decay (Table 4, Contrast 6).

Comparison of mites between the three body regions of pig cadavers (head, torso and posterior), and their corresponding control soils revealed that *Parasitus evertsi* and *M*. *matrius* were indicators for the posterior region, whereas *L*. *reticulata* was associated with the head. In the control soils *Q*. *michaeli*, *S*. *laevigatus* and *Hoplophora anomala* were all identified as indicators of the control plot C1 corresponding to ‘head’ sample (Table 4, Contrast 7).

## Discussion

### Abundance and successional patterns of mite groups during stages of decomposition

The mite fauna of soil changes in relation to different stages of decomposition (Bornemissza 1957). Fluctuations in mite populations and succession of species can be used as time proxies, in a similar manner to insects. This study is the first to show that buried cadavers cause a significant increase in mite abundance and diversity in the surrounding soil, attracting a unique composition of mites compared to the local (in situ) soil mite fauna, confirming that mites are an important part of the arthropod succession of buried cadavers. This is also the first study to show that there are successional patterns of mite fauna associated with each decomposition stage of buried cadavers highlighting their importance as forensic markers of the decay stages. Mesostigmata mites were the most abundant and diverse group of mites from cadaver soils followed by Oribatida, Prostigmata and Astigmata. This is not surprising as Mesostigmata mites are free-living and soil-dwelling mites, the majority of which are predaceous and play a fundamental role in decomposition processes of organic material such as carrion in forest soils (Koehler 1999; Krantz 1998). Many predatory Mesostigmata species are phoretic associates of necrophagous and necrophilous Diptera and Coleoptera and therefore an abundance on cadavers is expected throughout decay. Mesostigmatids were more dominant in cadaver soils compared to control soils and were absent in the fresh stages. The Mesostigmata collected from cadaver soils were mainly predatory species, either originating from different soil horizons or arriving via phoresy with necrophagous and necrophilous Diptera and Coleoptera, or with subterranean mammalian scavengers carrying their own phoretic mites (Perotti and Braig 2009).

As expected, the abundance of Mesostigmata mites increased as decomposition progressed. As a cadaver enters the bloated stage, volatile organic compounds (VOCs) are released into the atmosphere, and calliphorids (Diptera) detect and colonise the carcass. Many initial colonising flies will be carrying phoretic mites, which detach from their host organism soon after arrival to a cadaver and proceed to rapidly feed and reproduce (Perotti and Braig 2009; Perotti et al. 2009). This explains the presence of Mesostigmata mites during the bloated stages of all cadavers. As expected for buried cadavers, Diptera were seen ovipositing on the slightly exposed areas of the buried cadavers from the bloated stages onwards and the resulting larvae were seen rapidly moving deep, consuming the soft tissue. As this occurs, there is an increase in food availability resulting in an increased density of the cadaveric fauna (Carter et al. 2007; Goff 1993). Consequently, this attracts a myriad of predatory mite species from the surrounding soil and/or via phoresy explaining why mid-decomposition stages were associated with an overall highest abundance of mites.

In contrast to cadaver soils, Oribatida was the most abundant and diverse group of mites in control soils, followed by Mesostigmata, Prostigmata and Astigmata. Oribatida mites are mostly secondary decomposers, typically associated with soil litter and plant detritus and are expected to be the most abundant group of mites in most soil types as they are representatives of the ‘normal’ soil fauna (Norton and Ermilov 2014). The majority of Oribatida mites do not possess specialised dispersal stages such as those seen with Mesotigmata, Prostigmata and Astigmata and their dispersal abilities are limited (Krantz and Walter 2009). Whereas Oribatida mites were present throughout the decay of P1 and P2 they were always less abundant than Mesostigmata mites during bloated, active and advanced stages but more abundant during the fresh stage. Most do not have phoretic associations with necrophagous insects and, therefore, the oribatids associated with the cadavers were most likely to be those already present in the soil or opportunistic oribatids migrating from other soil horizons.

In cadavers, although predatory Mesostigmata mites dominate, soil-dwelling oribatids are thought to disappear due to predation or migration away from the cadaver in response to environmental changes in the soil—e.g., increased soil pH—soon after a cadaver is introduced (Bornemissza 1957). In contrast, the results of this study demonstrate that the Oribatida mites did not entirely disappear on introduction of a cadaver as there was a slight increase after fresh decay of P1 and P2 and they occurred, although in reduced numbers, throughout most of the stages. However, their abundance did fluctuate and their abundance was always lower compared to cadaver soils and they were entirely absent during bloated and active decay of P3, when Mesostigmata mites were the most abundant. Their lower abundances and temporal absence may have been because Oribatida mites are predated by Mesostigmata mites. Most Oribatida species are saprophages and mycophages, only a minority are opportunistic species and feed on nematodes and micro-organisms such as fungi and bacteria that occupy ephemeral habitats such as a decaying cadaver (Krantz 1978; Norton and Ermilov 2014), explaining why they were not entirely absent from cadaver soils. Interestingly, Oribatida mites increased in abundance during the late stages of decay. The decrease in Oribatida mites as well as other mites from the Astigmata and Prostigmata during the dry stage of P2 may be explained by the presence of a greater number of the predatory Mesostigmata species *Macrocheles matrius* during this stage, compared to the dry stage of cadaver 1 and 3.

Our results also demonstrate that soil pH had a significant and positive effect on the abundance of mites. Therefore, differences in soil pH from cadaver soils are likely to have contributed to the small variations in patterns of mite abundance between the pig cadavers, for example, mite abundance decreased during advanced decay in P1 and P3 when soil pH also decreased, whereas mite abundance increased in P2 during advanced decay when soil pH increased (alkaline). Certain mite groups are known to be sensitive to soil pH, with acidic conditions more favourable to Oribatida than Mesostigmata mites in forest soils, and Oribatida less abundant in alkaline soils (Maraun and Scheu 2000). This pattern was reflected in our results: in P1 and P3, as soil pH became less alkaline in advanced decay, the abundance of Mesostigmata mites decreased, whereas in P2 the alkalinity of the soil pH increased and an increase in Mesostigmata mites was recorded. The increment in Mesostigmata numbers in advanced decay of P2 was mainly a dominance of Parasitidae mites; many Mesostigmata families such as Parasitidae can thrive in moderately alkaline conditions (Manu et al. 2021). In P1 and P2, where the pH declined slightly, the abundance of Parasitidae from active to advanced remained the same. By contrast, during active and advanced decay of P1, soil pH was less alkaline than in P2 and P3 and interestingly, a slightly higher abundance of Oribatida mites was noted. This change may be the result of indirect effects on the density of food sources for Oribatida mites, as soil pH fluctuations influence densities of bacteria and fungi. Species of Oppiidae and Quadroppiidae, which feed on these micro-organisms, were present during late decomposition when the soil remained slightly alkaline for all cadavers.

Astigmata mites were recovered in relatively low abundance, being the least diverse mite group in cadaver and control soils. Their occurrence did not follow any successional pattern in response to decomposition. Prostigmata mites appeared to have some significant association with decay stages and were more abundant than in control soils. In terrestrial ecosystems, Prostigmata mites have diverse feeding methods in soils and are known to be phytophagous, saprophagous, parasitic, paraphagous with insects as well as predaceous. Prostigmata mites are not commonly thought to be significant members of the carrion fauna as only a small number of Prostigmata species is predaceous, whereas most are parasites and parasitoids of insects (Eickwort 1990). Prostigmata mites appeared to be mainly associated with bloated, active and advanced stages of decay. Their reduction/absence in the fresh stages and in control soils may suggest that they are just opportunistic. Some species of Prostigmata are phoretic on Phoridae, ‘coffin flies’ (Fain and Greenwood 1991; Perotti et al. 2009), flies that are common in graves (Martín‐Vega et al. 2011; Motter 1898) and may bring along these phoretic prostigmatids.

### Biodiversity of mites during stages of decomposition

In soils, the structure and dynamics of mite groups change in response to food availability and changes in environmental conditions (Behan-Pelletier 1999). The impact of vertebrate graves on the temporal succession and diversity of mite species is poorly understood; but variations in arthropod species richness in response to decay is known to occur in terrestrial environments (Schoenly and Reid 1987). The present study demonstrates that the biodiversity of mites in cadaver soils was higher during each decay stage compared to the corresponding control soils, apart from during the fresh stage (according to Shannon diversity index). Mite richness, diversity and evenness also fluctuated in control soils; however, this was likely a result of variations in spatial distribution of mites during the study, perhaps responding to seasonal effects, or temperature changes throughout the year.

In this study, active decay was the most species-rich and diverse decomposition stage (Shannon diversity index), whereas fresh decay was the least species-rich and least diverse stage. Biodiversity began increasing during the bloated stage, coincidentally with abundance of VOCs, produced through putrefactive processes attracting insects. Calliphorid eggs are a source of food and attract an abundance of predatory soil mites (Braig and Perotti 2009). Bloated stages showed the second highest diversities, confirming that shallow concealment still allows an adequate released of volatiles. As Diptera larvae develop during active decay, soft tissue breakdown is optimal and more VOCs are produced (Carter et al. 2007). This creates a highly nutrient-rich environment with ample food for a variety of arthropods, therefore active decomposition is associated with the greatest diversity of arthropods (Early and Goff 1986). Although the abundance of soil mites during the advanced and dry stage was still relatively high, the richness and evenness of species declined during these two stages. As the cadaver enters the advanced and dry stage, the amount of soft tissue present and arthropod activity is minimal and the food supply is gradually depleted resulting in a decline in faunal diversity (Carter et al. 2007), which is in line with the results of this study.

### Successional patterns of mite families and species during stages of decomposition

#### Mesostigmata families and species

In terrestrial environments Parasitidae, Macrochelidae, Ascidae and Uropodidae mites are the most common associates of necrophagous insects and cadaveric decay (Perez-Martinez et al. 2019; Perotti and Braig 2009). This partially agrees with the results of this study as Parasitidae and Macrochelidae were the most abundant and diverse mite families. Interestingly, Ascidae and Uropodidae mites were not significantly abundant in the grave environment. Non-phoretic and phoretic Parasitidae species, especially from the genera *Parasitus* and *Gamasodes*, associated with necrophagous insects have been recovered from various types of decomposition scenes and stages in terrestrial environments, mainly on the surface (González-Medina et al. 2013; Kamaruzaman et al. 2018; Perez-Martinez et al. 2019; Reed 1958; Saloña-Bordas et al. 2010; Saloña-Bordas and Perotti 2014).

In studies of graves, Parasitidae mites can colonise shallow as well as deep graves (Goff 1991; Rai et al. 2020; Vanlaerhoven and Anderson 1999). Parasitidae mites increased in abundance as the cadaver progressed from bloated, active and advanced decay and decreased during the dry stage. Their colonisation of cadavers during these stages is primarily due to the arrival of their phoretic hosts, as many deutonymphal Parasitidae are phoretic with necrophagous and necrophilous flies (Fain and Greenwood 1991; Hyatt 1980; Rai et al. 2020; Saloña-Bordas and Perotti 2019) and beetles (Costa 1963; González-Medina et al. 2013; Hyatt 1980; Schwarz and Walzl 1996; Schwarz and Müller 1992). This is reflected by the results of this study where they were significantly more abundant in cadaver soils, suggesting that most species associated with the shallow graves are phoretic Parasitidae.

*Cornigamasus lunaris* (Parasitidae, Mesostigmata) was significantly associated with cadaver soils, and was found to be an indicator species of the bloated stage. This species is known to rapidly inhabit ephemeral habitats such as decomposing plant matter, dung and compost across Europe (Witaliñski 2005). Phoretic deutonymphs have been recovered from necrophilous insects visiting carrion outdoors (Perez-Martinez et al. 2019) and from a human cadaver indoors during an unspecified stage of decay (Anderson 1995). In Europe, phoretic deutonymphs have been found on dung beetles (Kirk 1992) and residing in the nests and fur of subterranean rodents (Várfalvyová et al. 2011). Even though a few individuals were found in control soils, its significant association with cadaver soils may suggest that the prevalence of *C*. *lunaris* in graves is related to phoresy on coprophagous and coprophilous insects. This species is considered coprophilous, its arrival at the bloated stage may be due to phoresy on early-colonising insects, on visiting rodents attracted to the volatiles, or because the graves were near small mammal nests.

*Gamasodes spiniger* (Parasitidae, Mesostigmata) was not significant to any soil type, but was an indicator species of active decay. It is a phoretic of necrophagous Diptera and Coleoptera (*Copris hispanus*) (Costa 1963). Phoretic deutonymphs of *G*. *spiniger* were collected attached to lesser dung fly, the Spelobia fly (Sphaeroceridae) during active decay of two human corpses in a shallow grave concealed with manure (Rai et al. 2020). It is a known traveller on Sphaeroceridae flies (Lundqvist 1998, Samsinak 1989), its occurrence in graves is expected as Sphaeroceridae are extremely common due to their ability to access the cadaver via small crevices in the soil (Pastula and Merritt 2013). *Gamasodes spiniger* is coprophilous, its affinity to active decay might be a result of the purging of internal fluids occurring during this stage (which includes faecal material). *Gamasodes spiniger* has been documented from surface carrion during different stages of decomposition (Anderson and VanLaerhoven 1996; Lundqvist 1998), suggesting that in graves this species is a strong marker of active decay as a result of phoretic arrival with small lesser dung flies attracted to decay fluids containing faecal material. Its association with both cadaver and control soils (though in greater abundance in cadaver soils) suggests that it can colonise shallow graves from the surrounding soil as well as phoretically with Diptera.

*Vulgarogamasus remberti* (Parasitidae, Mesostigmata) was found to be significantly associated with cadaver soil, but no significant association was found with decay stage, body region or a pig cadaver subject. This species is especially common in the fur and nests of above- and below-ground animals such as birds and moles (Hyatt 1980; Mašán and Stanko 2005). There is only one study documenting its occurrence with surface decay, attributing its phoretic arrival by visiting shrews (Perez-Martinez et al. 2019). Cadavers buried in graves of less than 30 cm deep are not entirely protected from small mammalian scavengers such as rodents (Rodriguez and Bass 1985). Small rodents can access cadavers in shallow graves via the surface or through subterranean channels and bring along predatory phoretic mites associated with their fur or species that reside in their nests. The colonisation of *V*. *remberti* and other *Vulgarogamasus* species found in the cadaver soils (*V*. *kraeplini* and *V*. *oudemansi*) is also likely due to phoretic arrival with subterranean small mammals visiting the cadavers for food.

*Parasitus evertsi* (Parasitidae, Mesostigmata) was significantly associated with the posterior region, with no association with either soil type or stage of decomposition. In England *P*. *evertsi* inhabits caves, tree holes, fur of shrews and other small mammals, soil and fungi (Hyatt 1980). It has been recovered from Yew (*Taxus bacata*) in England (Skorupski and Luxton 1998). Its association with cadavers is likely to be incidental as this species is already native to forest soils where it colonises decaying vegetation rather than carrion (Skorupski and Luxton 1998).

*Eugamasus* sp. (Parasitidae, Mesostigmata) was a significant indicator of advanced decay and it was specifically associated with the advanced decomposition of P2. This genus has phoretic associations with beetles (Moser and Roton 1971), its absence from control soils suggests arrival to the cadaver via phoretic dispersal perhaps from beetles rather than from the surrounding soil. *Eugamasus* colonization may be concurrent with occurrence of nematodes, which are known to increase in density and diversity during active, advanced and dry stages (Szelecz et al. 2016). Well studied species as *E*. *cavernicolus* inhabit caves, tunnels and nests of subterraneous mammals (Fenďa and Lukáš 2014).

The second most abundant and species-rich mite family in cadaver soils was Macrochelidae. Macrochelidae species have been recovered from cadavers during all stages of decay, in exposed (Goff 1989; Kamaruzaman et al. 2018; Saloña-Bordas et al. 2010; Szelecz et al. 2018) as well as buried cadavers (Anderson and VanLaerhoven 1996; Goff 1991; Kamaruzaman et al. 2018; Rysavy and Goff 2015; VanLaerhoven and Anderson 1999). Their high abundance in cadaver soils is not surprising as Macrochelidae species are predatory and are involved in the decomposition processes of ephemeral organic matter such as carrion, dung, compost and other organic materials. They prey on successive waves of Diptera and Coleoptera eggs and larvae and nematodes as well as a variety of micro-arthropods including Collembola (springtails) and other mites (Krantz 1998).

Macrochelidae mites may visit cadavers throughout decomposition from the early to the dry stages as female Macrochelidae mites are phoretic with necrophagous flies and carrion beetles (Barton et al. 2014; Perotti and Braig 2009; Perotti et al. 2009; Saloña-Bordas and Perotti 2015). Female Macrochelidae mites are amongst the first mites to colonise exposed cadavers in early stages with necrophagous Diptera (Early and Goff 1986; Goff 1989; Perotti and Braig 2009; Perotti et al. 2009; Rysavy and Goff 2015). The time of cadaver colonisation by Macrochelidae mites is dependent on the species, as some species are phoretic on early-colonising Diptera and will colonise during the early stages, whereas other species are phoretic on late-colonising Coleoptera and may arrive at later stages and feed on the eggs of flies (Early and Goff 1986; Goff 1991; Leclercq and Verstraeten 1988; Reed 1958). Colonisation patterns of *Macrocheles* mites in particular have complemented information on the time of death in case studies (Goff 1991; Kamaruzaman et al. 2018; Szelecz et al. 2018). In this study, they colonised at the bloated stages, likely to have arrived with early-colonising insects and decline in numbers during active and advanced decay, perhaps due to competition with a greater abundance of predatory Parasitidae mites. Their re-appearance in abundance at dry stages concurs with a reduction in Parasitidae. This study demonstrates that in graves Macrochelidae mites appear to colonise mainly during later stages of decay rather than early stages such as seen on surface decomposition.

*Macrocheles* is the most diverse genus of this family and the majority of *Macrocheles* species colonise cadavers through phoresy by virgin females, attaching to necrophagous and necrophilous Diptera and Coleoptera (Kamaruzaman et al. 2018). *Macrocheles matrius* was the most abundant in the present study. It most commonly inhabits poultry litter and farms and granaries, and is thought to be exogenous to forest soils but may exist in some forest habitats. In this study, it was a marker of cadaver soil and of the dry/remains stage, and was associated with the posterior body region. *Macrocheles matrius* has been found in the soil up to 10 cm deep beneath the exposed and dry human bones in a forest, agreeing with its association with the dry stage (Szelecz et al. 2018). It is a species mainly phoretic on mammals (Krantz and Whitaker 1988) and has been recovered from the nests of mound-building mice (Mašán and Stanko 2005). It may utilise small mammals to locate and colonise cadavers. Importantly, *M*. *matrius* has already been found utilising cadaver-associated dung beetles such as *Geotrupes silvaticus* for phoretic dispersal (Hughes 1976) which locates the beetles and the mites in the posterior area of the body, near the anus. In crime scenes, access to specific body parts by insects carrying mites, and thus occurrence of species-specific phoretic mites, can add information on localization of wounds, helping describe the circumstances related to death. For example, if the victim was attacked and/or where and which of the injuries might have caused the death; or, what type of attack was performed, was the victim raped? The presence of this species in the cadaver soils and colonisation during the dry stage is likely to be in accordance with the presence of Diptera and Coleoptera eggs and larvae as well as other mites which this species predates on (Soliman et al. 1978). The association with the body posterior region may explain its phoretic arrival with dung beetles, attracted to the volatiles from the faecal matter released with decay fluids from the anal region during active decomposition (Kamaruzaman et al. 2018).

Other Mesostigmata families colonised the cadavers in lower numbers throughout decomposition, such as Diagamasellidae, Pachylaelapidae, Ascidae, Diathrophallidae, Uropodidae, and Protodinychidae. Digamasellidae species are mainly predatory and feed on cadaveric fauna such as Diptera eggs and early instars, nematodes, fungi and Collembola (Walter et al. 1988), and deutonymphs are phoretic with necrophagous flies such as Muscidae (Pereira Sato et al. 2018). Muscidae flies are common in graves due to their smaller size compared to larger calliphorids (Gaudry 2010). Pachylaelapidae have been collected from a human cadaver in a shallow soil grave undergoing skeletisation (Goff 1991). In our study, *Pachylaelaps longisetis* was not significantly associated with cadaver soils but was significantly associated with only C2 (in two samples). This species has a patchy distribution in forest soils and is an unlikely marker of the grave fauna. Members of *Pachylaelaps* may occur in shallow graves incidentally, as *P*. *longisetis* has been recovered from bare soil samples up to a depth of 5 cm from spruce forests (Skorupski et al. 2009) and patchy distributions of soil organisms can result from varying soil organic matter content (Fromm et al. 1993).

Ascidae mites occurred during every stage of decay in low numbers (> 5 individuals) except in the fresh stage, suggesting that Ascidae mites are attracted to decomposition in shallow graves. Ascidae mites occupy a wide range of microhabitats in forest soils from the subsoil to leaf litter, and in dry as well as wet environments (Kalúz and Fenďa 2005), explaining their occurrence during the bloated, active, advanced and the dry stages. These mites are phoretic with Diptera of forensic importance (Perotti and Braig 2009). Ascidae mites have been recovered from exposed cadavers in past studies in forest soils (Perez-Martinez et al. 2019; Saloña-Bordas et al. 2010). This is their first report from graves. Diarthrophallidae mites have an exclusive commensal association with Passalidae beetles (Krantz and Walter 2009). They were found in low numbers (< 5 individuals) during active and advanced decay and were absent in control soils suggesting that their carriers were attracted to decomposition.

In graves, Uropodidae mites appear to be common during the skeletonised stages. For example, mites from the infra-order Uropodina (includes the family Uropodidae) were collected from buried skeletonised human remains (Goff 1991; Mariani et al. 2014), whereas *Uropoda depressa* (Uropodidae) was amongst the most abundant species collected from human graves by Motter (1898). The results of this study showed only one Uropodidae mite found during the active stage and one during and advanced stage of P2. Likewise, only two individuals of Protodinychidae were recovered in the bloated stage of P1 and were absent in control soils. The reason of their absence during this study could be due to this study finishing at the beginning of skeletal remains. Thus, their absence may suggest association with later skeletal remains, rather than with early stages. We cannot ignore that there might be attraction of Uropodidae to human cadavers, as this experiment was conducted with pig-carcasses.

#### Oribatida families and species

After introduction of the cadavers, there was disappearance of some incidental Oribatida mites such as fungivorous families Damaeidae and Suctobelbidae (Miko and Mourek 2008). These oribatids are likely to have moved away from the cadaver soon after its introduction due to changes in soil pH that led to a more alkaline soil environment. *Scheloribates laevigatus* (Scheloribatidae) was an indicator of control soils. *Scheloribates laevigatus* are soil-dwelling mites which inhabit the surface of soils residing in moss, humus and decaying wood and are microbial feeders (Hughes 1976). Until now its association with cadavers was almost unnoticed, but they might feed on microbes associated with decay in graves (Anderson and VanLaerhoven 1996). Two species of Oribatida were found to be significantly associated with the dry stages of P1 only: *Quadroppia michaeli* (Quadroppidae) and *Ramusella clavipectinata* (Oppidae). Colonisation during the dry stages may be due to the soil conditions normalising towards the end stages (Mariani et al. 2014; Walter and Proctor 1999). Their low numbers or absence in all other samples might suggest a patchy spatial dispersal in forest soil. Oribatida mites are known to exhibit specific spatial distributions in soil habitats due to their limited ability to disperse and their association to soil parameters such as microbial biomass and organic matter content (Minor 2011). *Quadroppia michaeli* and *R*. *clavipectinata* are fungivores and association to dry stages may result by attraction to fungal growth on bones (Hawksworth and Wiltshire 2011). The Oppiidae family is one of the very few Oribatida groups that display phoresy with beetles (Norton 1980), and *R*. *clavipectinata* has been found to be phoretically associated with Curculionoidea beetles (Ermilov and Frolov 2019). This species may have arrived with beetles in the dry stage to exploit the characteristic fungal growth on the bones at this stage.

#### Prostigmata families and species

This is the first study to highlight the occurrence of Prostigmata mites in graves. Most Prostigmata were Tydeidae and they were mainly associated with advanced decay, represented by a single species, *Lorryia reticulata*. *Lorryia reticulata* was a marker of cadaver soils, of advanced decay and P3. Until now, its association with decay, especially in graves was unknown. It was also significantly abundant in C3, in the samples taken while P3 was bloated. This family of mites has extremely diverse feeding strategies although most feed on algae, fungi and pollen. Tydeids occupy a large variety of micro-habitats within forests from soil litter, plant detritus, bark and hollows of trees to bird nests (Kaźmierski et al. 2018). Phoresy is uncommon in this family (Treat 1975). The significant association with the advanced decay is likely to have been opportunistic, arriving from the surrounding soil layers in order to feed on associated mites and their eggs, nematodes and fungi.

## Conclusion

The research presented here allows for a greater understanding of the colonisation patterns of mite species and their value as markers of decay stages in shallow graves. This study has shown that an abundance of mite groups colonize shallowly buried cadavers in successive patterns following stages of decay, and Mesostigmata mites are the most dominant and diverse mite group. Despite the delays experienced by insects in finding a grave, phoretic species arriving with insect or mammal hosts formed the majority of the mite fauna of shallow graves. In particular, phoretic Parasitidae appear to be markers of bloated, active and advanced stages, whereas phoretic Macrochelidae were markers of the dry stages, along with an unexpected diversity of Oribatida and Prostigmata mites. This study has also shown that specific mite species may be associated with cadaver body regions; particularly in cases where the mites have been brought to the cadaver (phoresy) by carriers arriving and colonising specific parts of the body. One good example are Fannidae manure flies and Geotrupidae dung beetles, which can bring macrochelids to the posterior part of the body where faeces can become exposed.

## Supplementary Information

Below is the link to the electronic supplementary material.

Supplementary file1 (DOCX 24 KB)

Supplementary file2 (DOCX 27 KB)

Supplementary file3 (DOCX 20 KB)

Supplementary file4 (DOCX 23 KB)

Supplementary file5 (DOCX 20 KB)

Supplementary file6 (DOCX 18 KB)

## Data Availability

All data is made available in tables in the main text/manuscript and in the Supplementary Materials file.

## Declarations

### Permit to work with livestock carcasses

Provided in Materials and Methods (APHA-TARP).
