# Supplementary material for: Assemblages of Acari in shallow burials: mites as markers of the burial environment, of the stage of decay and of body-cadaver regions
Source: Exp Appl Acarol. 2021 Oct 7;85(2-4):247–76. doi: 10.1007/s10493-021-00663-x (PMC8604864; doi:10.1007/s10493-021-00663-x)
Supplement: Supplementary file 1 — Supplementary file1 (DOCX 24 KB) [file 10493_2021_663_MOESM1_ESM.docx]

ONLINE RESOURCE 1

**Experimental and Applied Acarology**

**Assemblages of Acari of shallow burials: mites as markers of the burial environment, of the stage of decay and of body-cadaver regions.**

Jas K. Rai, Brian J. Pickles, M. Alejandra Perotti

Ecology and Evolutionary Biology Section, School of Biological Sciences, University of Reading, Reading, Berkshire, UK

Corresponding author:

M. Alejandra Perotti

[m.a.perotti@reading.ac.uk](mailto:m.a.perotti@reading.ac.uk)

**Figure S1: Preliminary study of mite diversity in cadaver soils.** The number of mite species collected from varying soil samples sizes (1, 2, 3, and 4 dl) from beneath four surface pig cadavers

**Figure S2: Preliminary study of mite diversity in control soils.** The number of mite species collected from varying soil samples sizes (1, 2, 3, and 4 dl) from bare surface forest soil.

.

**Table S1: Preliminary study of mite diversity in cadaver soils.** The number of mite species collected from varying soil samples sizes (1, 2, 3, and 4 dl) from beneath four surface pig cadavers.

|  | 1 dl | 2 dl | 3 dl | 4 dl |
| --- | --- | --- | --- | --- |
| Pig 1 | 10 | 13 | 10 | 6 |
| Pig 2 | 12 | 15 | 14 | 12 |
| Pig 3 | 13 | 10 | 12 | 12 |
| Pig 4 | 8 | 15 | 16 | 10 |

**Table S2: Preliminary study of mite diversity in control soils.** The number of mite species collected from varying control soil samples sizes (1, 2, 3, and 4 dl) from bare surface forest soil.

|  | 1 dl | 2 dl | 3 dl | 4 dl |
| --- | --- | --- | --- | --- |
| Control 1 | 5 | 9 | 10 | 9 |
| Control 2 | 3 | 9 | 7 | 8 |
| Control 3 | 1 | 5 | 3 | 3 |
| Control 4 | 4 | 5 | 8 | 5 |
