# Supplementary material for: Assemblages of Acari in shallow burials: mites as markers of the burial environment, of the stage of decay and of body-cadaver regions
Source: Exp Appl Acarol. 2021 Oct 7;85(2-4):247–76. doi: 10.1007/s10493-021-00663-x (PMC8604864; doi:10.1007/s10493-021-00663-x)
Supplement: Supplementary file 2 — Supplementary file2 (DOCX 27 KB) [file 10493_2021_663_MOESM2_ESM.docx]

ONLINE RESOURCE 2

**Experimental and Applied Acarology**

**Assemblages of Acari of shallow burials: mites as markers of the burial environment, of the stage of decay and of body-cadaver regions.**

Jas K. Rai, Brian J. Pickles, M. Alejandra Perotti

Ecology and Evolutionary Biology Section, School of Biological Sciences, University of Reading, Reading, Berkshire, UK

Corresponding author:

M. Alejandra Perotti

[m.a.perotti@reading.ac.uk](mailto:m.a.perotti@reading.ac.uk)

**Supplementary Table S3:** The pH of three soil samples collected during each stage of decomposition of each pig cadaver P1, P2 and P3, the average soil pH during each stage and the standard error of means (+/-).

| **Stage of decomposition** | **Fresh** | **Bloated** | **Active** | **Advanced** | **Dry** |
| --- | --- | --- | --- | --- | --- |
| P1 | 7.3 | 8.1 | 8.0 | 9.1 | 8.0 |
|  | 7.2 | 8.2 | 8.8 | 8.3 | 8.1 |
|  | 7.2 | 8.2 | 9.1 | 8.2 | 8.1 |
| Average | 7.2 | 8.2 | 8.6 | 8.5 | 8.1 |
| Standard error (+/-) | 0.033 | 0.033 | 0.328 | 0.285 | 0.033 |
| P2 | 7.2 | 8.7 | 7.9 | 8.8 | 8.2 |
|  | 8.5 | 8.5 | 8.0 | 8.6 | 8.2 |
|  | 8.4 | 7.9 | 8.8 | 8.5 | 8.1 |
| Average | 8.0 | 8.4 | 8.2 | 8.6 | 8.2 |
| Standard error (+/-) | 0.418 | 0.240 | 0.285 | 0.088 | 0.033 |
| P3 | 7.1 | 7.2 | 8.7 | 8.5 | 8.2 |
|  | 7.0 | 8.8 | 8.7 | 8.5 | 7.2 |
|  | 7.0 | 8.7 | 8.6 | 8.0 | 8.0 |
| Average | 7.0 | 8.2 | 8.7 | 8.3 | 7.8 |
| Standard error (+/-) | 0.033 | 0.517 | 0.033 | 0.167 | 0.306 |

**Supplementary Table S4:** GLM analysis of the interactive effects of treatment (pig cadaver, control) and decay stage (fresh, bloated, active, advanced, dry/remains) on soil pH during decomposition in replicates 1-3. Bold text indicates significant model terms (P < 0.05).

| **Replicate** | **Factor** | **Estimate** | **St.Error** | **t value** | **P value** | **Model R^2^** |
| --- | --- | --- | --- | --- | --- | --- |
| P1 vs C1 | **(Intercept)** | **6.133** | **0.215** | **28.56** | **< 0.001** | 0.877 |
|  | **Treatment-Pig** | **1.100** | **0.304** | **3.62** | **0.002** |  |
|  | **Stage-Bloated** | **0.733** | **0.304** | **2.42** | **0.025** |  |
|  | **Stage-Active** | **0.967** | **0.304** | **3.18** | **0.005** |  |
|  | **Stage-Advanced** | **0.967** | **0.304** | **3.18** | **0.005** |  |
|  | Stage-Dry | 0.467 | 0.304 | 1.54 | 0.140 |  |
|  | Pig:Bloated | 0.200 | 0.430 | 0.47 | 0.646 |  |
|  | Pig:Active | 0.433 | 0.430 | 1.01 | 0.325 |  |
|  | Pig:Advanced | 0.333 | 0.430 | 0.78 | 0.447 |  |
|  | Pig:Dry | 0.367 | 0.430 | 0.85 | 0.403 |  |
| P2 vs C2 | **(Intercept)** | **7.433** | **0.200** | **37.12** | **< 0.001** | 0.749 |
|  | **Treatment-Pig** | **6.000** | **0.283** | **2.12** | **0.047** |  |
|  | Stage-Bloated | < -0.001 | 0.283 | 0.00 | 1.000 |  |
|  | Stage-Active | 0.067 | 0.283 | 0.24 | 0.816 |  |
|  | Stage-Advanced | -0.033 | 0.283 | -0.12 | 0.908 |  |
|  | Stage-Dry | -0.333 | 0.283 | -1.18 | 0.253 |  |
|  | Pig:Bloated | 0.333 | 0.401 | 0.83 | 0.415 |  |
|  | Pig:Active | 0.133 | 0.401 | 0.33 | 0.743 |  |
|  | Pig:Advanced | 0.633 | 0.401 | 1.58 | 0.130 |  |
|  | Pig:Dry | 0.467 | 0.401 | 1.17 | 0.258 |  |
| P3 vs C3 | **(Intercept)** | **7.000** | **0.206** | **33.98** | **< 0.001** | 0.811 |
|  | Treatment-Pig | 0.033 | 0.291 | 0.11 | 0.910 |  |
|  | Stage-Bloated | 0.033 | 0.291 | 0.11 | 0.910 |  |
|  | Stage-Active | 0.333 | 0.291 | 1.14 | 0.266 |  |
|  | Stage-Advanced | 0.133 | 0.291 | 0.46 | 0.652 |  |
|  | Stage-Dry | 0.100 | 0.291 | 0.34 | 0.735 |  |
|  | **Pig:Bloated** | **1.167** | **0.412** | **2.83** | **0.010** |  |
|  | **Pig:Active** | **1.300** | **0.412** | **3.16** | **0.005** |  |
|  | **Pig:Advanced** | **1.167** | **0.412** | **2.83** | **0.010** |  |
|  | Pig:Dry | 0.667 | 0.412 | 1.62 | 0.121 |  |
