# Supplementary material for: Assemblages of Acari in shallow burials: mites as markers of the burial environment, of the stage of decay and of body-cadaver regions
Source: Exp Appl Acarol. 2021 Oct 7;85(2-4):247–76. doi: 10.1007/s10493-021-00663-x (PMC8604864; doi:10.1007/s10493-021-00663-x)
Supplement: Supplementary file 3 — Supplementary file3 (DOCX 20 KB) [file 10493_2021_663_MOESM3_ESM.docx]

ONLINE RESOURCE 3

**Experimental and Applied Acarology**

**Assemblages of Acari of shallow burials: mites as markers of the burial environment, of the stage of decay and of body-cadaver regions.**

Jas K. Rai, Brian J. Pickles, M. Alejandra Perotti

Ecology and Evolutionary Biology Section, School of Biological Sciences, University of Reading, Reading, Berkshire, UK

Corresponding author:

M. Alejandra Perotti

[m.a.perotti@reading.ac.uk](mailto:m.a.perotti@reading.ac.uk)

**Supplementary Table S5:** The median number of mites from the 4 major mite Orders (Mesostigmata, Oribatida, Astigmata and Prostigmata) associated with each stage of decomposition of pig cadavers (n=3) (fresh, bloated, active, advanced and dry remains) and the Kruskal-Wallis test (adjusted for ties) showing that there was no statistical significance in the abundance of either mite Order associated with either stage of decomposition (P>0.05).

| **Mite Order** | **Stage of decomposition** | **Median** | **Mean Rank** | **Overall Rank** | **Z value** | **H value** | **P-value (adjusted for ties)** |
| --- | --- | --- | --- | --- | --- | --- | --- |
| Mesostigmata | Fresh | 0 | 2.0 | 8.0 | -2.60 | 7.29 | 0.12 |
| Mesostigmata | Bloated | 9 | 9.3 |  | 0.58 |  |  |
| Mesostigmata | Active | 14 | 10.5 |  | 1.08 |  |  |
| Mesostigmata | Advanced | 6 | 8.2 |  | 0.07 |  |  |
| Mesostigmata | Dry/ remains | 11 | 10.0 |  | 0.87 |  |  |
| Oribatida | Fresh | 1 | 4.5 | 8.0 | -1.52 | 3.04 | 0.55 |
| Oribatida | Bloated | 2 | 7.3 |  | -0.29 |  |  |
| Oribatida | Active | 3 | 8.5 |  | 0.22 |  |  |
| Oribatida | Advanced | 3 | 9.5 |  | 0.65 |  |  |
| Oribatida | Dry/ remains | 4 | 10.2 |  | 0.94 |  |  |
| Astigmata | Fresh | 0 | 4.5 | 8.0 | -1.52 | 4.19 | 0.38 |
| Astigmata | Bloated | 0 | 6.8 |  | -0.51 |  |  |
| Astigmata | Active | 1 | 8.5 |  | 0.22 |  |  |
| Astigmata | Advanced | 1 | 9.3 |  | 0.58 |  |  |
| Astigmata | Dry/ remains | 3 | 10.8 |  | 1.23 |  |  |
| Prostigmata | Fresh | 0 | 3.5 | 8.0 | -1.95 | 5.81 | 0.21 |
| Prostigmata | Bloated | 2 | 7.7 |  | -0.14 |  |  |
| Prostigmata | Active | 5 | 9.8 |  | 0.79 |  |  |
| Prostigmata | Advanced | 8 | 11.5 |  | 1.52 |  |  |
| Prostigmata | Dry/ remains | 3 | 7.5 |  | -0.22 |  |  |
