# Supplementary material for: Assemblages of Acari in shallow burials: mites as markers of the burial environment, of the stage of decay and of body-cadaver regions
Source: Exp Appl Acarol. 2021 Oct 7;85(2-4):247–76. doi: 10.1007/s10493-021-00663-x (PMC8604864; doi:10.1007/s10493-021-00663-x)
Supplement: Supplementary file 4 — Supplementary file4 (DOCX 23 KB) [file 10493_2021_663_MOESM4_ESM.docx]

ONLINE RESOURCE 4

**Experimental and Applied Acarology**

**Assemblages of Acari of shallow burials: mites as markers of the burial environment, of the stage of decay and of body-cadaver regions.**

Jas K. Rai, Brian J. Pickles, M. Alejandra Perotti

Ecology and Evolutionary Biology Section, School of Biological Sciences, University of Reading, Reading, Berkshire, UK

Corresponding author:

M. Alejandra Perotti

[m.a.perotti@reading.ac.uk](mailto:m.a.perotti@reading.ac.uk)

**Supplementary Table S6:** The successional patterns of mite families in control soils collected throughout the five stages of decomposition of the pig cadavers (n=3).

| Key | | | | | | | | |
| --- | --- | --- | --- | --- | --- | --- | --- | --- |
| Total number of mites | 0 | 1-5 | 6-10 | 11-15 | 16-20 | 21-25 | 26-30 | 31-35 |
| Colour |  |  |  |  |  |  |  |  |

| Order | Family | Control  Fresh | Control  Bloated | Control  Active | Control  Advanced | Control  Dry/ remains |
| --- | --- | --- | --- | --- | --- | --- |
| Mesostigmata | Parasitidae | 0 |  |  |  |  |
|  | Macrochelidae | 0 |  | 0 |  |  |
|  | Digamasellidae |  |  |  |  |  |
|  | Ascidae | 0 | 0 | 0 |  |  |
|  | Nothogynidae | 0 | 0 | 0 |  |  |
|  | Diarthrophallidae |  |  |  |  |  |
|  | Uropodidae |  |  |  |  |  |
|  | Pachylaelapidae | 0 |  | 0 |  |  |
|  | Laelapidae | 0 | 0 | 0 |  |  |
|  | Protodinychidae |  |  |  |  |  |
| Oribatida | Nothridae |  |  |  |  |  |
|  | Quadroppiidae |  |  |  |  |  |
|  | Damaeidae |  |  |  |  |  |
|  | Scheloribatidae |  |  |  |  |  |
|  | Oppiidae |  |  |  |  |  |
|  | Micreremidae |  |  |  |  |  |
|  | Euphthiracaridae |  |  |  |  |  |
|  | Achipteridae |  |  |  |  |  |
|  | Suctobelbidae |  |  |  |  |  |
|  | Eniochthoniidae |  |  |  |  |  |
|  | Eremulidae |  |  |  |  |  |
|  | Eremaeidae |  |  |  |  |  |
|  | Megermaeidae |  |  |  |  |  |
|  | Phthiracaridae |  |  |  |  |  |
|  | Galumnidae |  |  |  |  |  |
|  | Chamobatidae |  |  |  |  |  |
| Astigmata | Suidasiidae |  |  |  |  |  |
|  | Acaridae |  |  |  |  |  |
|  | Pyroglyphidae |  |  |  |  |  |
|  | Chortoglyphidae |  |  |  |  |  |
|  | Lemanniellidae |  |  |  |  |  |
|  | Scatoglyphidae |  |  |  |  |  |
| Prostigmata | Iolinidae |  |  |  |  |  |
|  | Ereynetidae |  |  |  |  |  |
|  | Eupodidae |  |  |  |  |  |
|  | Tydeidae |  |  |  |  |  |
|  | Rhagidiidae |  |  |  |  |  |
