# Supplementary material for: Assemblages of Acari in shallow burials: mites as markers of the burial environment, of the stage of decay and of body-cadaver regions
Source: Exp Appl Acarol. 2021 Oct 7;85(2-4):247–76. doi: 10.1007/s10493-021-00663-x (PMC8604864; doi:10.1007/s10493-021-00663-x)
Supplement: Supplementary file 5 — Supplementary file5 (DOCX 20 KB) [file 10493_2021_663_MOESM5_ESM.docx]

ONLINE RESOURCE 5

**Experimental and Applied Acarology**

**Assemblages of Acari of shallow burials: mites as markers of the burial environment, of the stage of decay and of body-cadaver regions.**

Jas K. Rai, Brian J. Pickles, M. Alejandra Perotti

Ecology and Evolutionary Biology Section, School of Biological Sciences, University of Reading, Reading, Berkshire, UK

Corresponding author:

M. Alejandra Perotti

[m.a.perotti@reading.ac.uk](mailto:m.a.perotti@reading.ac.uk)

**Supplementary Table S7:** The median number of 4 most abundant mite families (Parasitidae, Macrochelidae, Quadroppiidae and Tydeidae) associated with each stage of decomposition of pig cadavers (n=3) (fresh, bloated, active, advanced and dry/ remains) and the Kruskal-Wallis test showing that only the abundance of Parasitidae mites between decomposition stages is statistically significant (P>0.05, adjusted for ties).

| Mite family | Stage of decomposition | Median | Mean Rank | Overall Rank | Z value | H value | P-value (adjusted for ties) |
| --- | --- | --- | --- | --- | --- | --- | --- |
| Parasitidae | Fresh | 0 | 2.0 | 8.0 | -2.60 | 9.60 | 0.04 |
| Parasitidae | Bloated | 9 | 12.0 |  | 1.73 |  |  |
| Parasitidae | Active | 5 | 10.3 |  | 1.01 |  |  |
| Parasitidae | Advanced | 5 | 9.5 |  | 0.65 |  |  |
| Parasitidae | Dry/ remains | 2 | 6.2 |  | -0.79 |  |  |
| Macrochelidae | Fresh | 0 | 4.0 | 8.0 | -1.73 | 7.96 | 0.09 |
| Macrochelidae | Bloated | 0 | 7.3 |  | -0.29 |  |  |
| Macrochelidae | Active | 1 | 10.3 |  | 1.01 |  |  |
| Macrochelidae | Advanced | 0 | 5.8 |  | -0.94 |  |  |
| Macrochelidae | Dry/ remains | 5 | 12.5 |  | 1.95 |  |  |
| Quadroppiidae | Fresh | 0 | 5.5 | 8.0 | -1.08 | 3.15 | 0.53 |
| Quadroppiidae | Bloated | 0 | 8.0 |  | 0.00 |  |  |
| Quadroppiidae | Active | 0 | 8.3 |  | 0.14 |  |  |
| Quadroppiidae | Advanced | 0 | 7.3 |  | -0.29 |  |  |
| Quadroppiidae | Dry/ remains | 2 | 10.8 |  | 1.23 |  |  |
| Tydeidae | Fresh | 0 | 4.5 | 8.0 | -1.52 | 6.27 | 0.18 |
| Tydeidae | Bloated | 0 | 7.5 |  | -0.22 |  |  |
| Tydeidae | Active | 0 | 7.0 |  | -0.43 |  |  |
| Tydeidae | Advanced | 7 | 12.7 |  | 2.02 |  |  |
| Tydeidae | Dry/ remains | 1 | 8.3 |  | 0.14 |  |  |
