# Supplementary material for: Assemblages of Acari in shallow burials: mites as markers of the burial environment, of the stage of decay and of body-cadaver regions
Source: Exp Appl Acarol. 2021 Oct 7;85(2-4):247–76. doi: 10.1007/s10493-021-00663-x (PMC8604864; doi:10.1007/s10493-021-00663-x)
Supplement: Supplementary file 6 — Supplementary file6 (DOCX 18 KB) [file 10493_2021_663_MOESM6_ESM.docx]

ONLINE RESOURCE 6

**Experimental and Applied Acarology**

**Assemblages of Acari of shallow burials: mites as markers of the burial environment, of the stage of decay and of body-cadaver regions.**

Jas K. Rai, Brian J. Pickles, M. Alejandra Perotti

Ecology and Evolutionary Biology Section, School of Biological Sciences, University of Reading, Reading, Berkshire, UK

Corresponding author:

M. Alejandra Perotti

[m.a.perotti@reading.ac.uk](mailto:m.a.perotti@reading.ac.uk)

**Supplementary Table S8:** The species richness, Shannon diversity index (H) and evenness of species (E) associated with decomposition stages of cadavers (n=3).

| Index | Fresh | Bloated | Active | Advanced | Dry/ remains |
| --- | --- | --- | --- | --- | --- |
| Species richness (S) | 2 | 27 | 38 | 27 | 30 |
| Shannon (H) | 0.64 | 2.96 | 3.27 | 2.77 | 2.57 |
| Evenness (E) | 0.95 | 0.71 | 0.69 | 0.59 | 0.43 |

**Supplementary Table S9:** The species richness, Shannon diversity index (H) and evenness of species (E) associated with corresponding control soils.

| Index | Control Fresh | Control Bloated | Control Active | Control Advanced | Control Dry/ remains |
| --- | --- | --- | --- | --- | --- |
| Species richness (S) | 5 | 13 | 16 | 12 | 18 |
| Shannon (H) | 1.49 | 2.23 | 2.62 | 2.02 | 2.56 |
| Evenness (E) | 0.89 | 0.71 | 0.86 | 0.63 | 0.72 |
